# Sensing Techniques for Structural Health Monitoring: A State-of-the-Art Review on Performance Criteria and New-Generation Technologies

**DOI:** 10.3390/s25051424

**Published:** 2025-02-26

**Authors:** Ali Mardanshahi, Abhilash Sreekumar, Xin Yang, Swarup Kumar Barman, Dimitrios Chronopoulos

**Affiliations:** Department of Mechanical Engineering & Mecha(tro)nic System Dynamics (LMSD), KU Leuven, 9000 Gent, Belgium; abhilash.sreekumar@kuleuven.be (A.S.); swarupkumar.barman@kuleuven.be (S.K.B.); dimitrios.chronopoulos@kuleuven.be (D.C.)

**Keywords:** structural health monitoring, damage assessment, sensing techniques, performance criteria

## Abstract

This systematic review examines the capabilities, challenges, and practical implementations of the most widely utilized and emerging sensing technologies in structural health monitoring (SHM) for infrastructures, addressing a critical research gap. While many existing reviews focus on individual methods, comprehensive cross-method comparisons have been limited due to the highly tailored nature of each technology. We address this by proposing a novel framework comprising five specific evaluation criteria—deployment suitability in SHM, hardware prerequisites, characteristics of the acquired signals, sensitivity metrics, and integration with Digital Twin environments—refined with subcriteria to ensure transparent and meaningful performance assessments. Applying this framework, we analyze both the advantages and constraints of established sensing technologies, including infrared thermography, electrochemical sensing, strain measurement, ultrasonic testing, visual inspection, vibration analysis, and acoustic emission. Our findings highlight critical trade-offs in scalability, environmental sensitivity, and diagnostic accuracy. Recognizing these challenges, we explore next-generation advancements such as self-sensing structures, unmanned aerial vehicle deployment, IoT-enabled data fusion, and enhanced Digital Twin simulations. These innovations aim to overcome existing limitations by enhancing real-time monitoring, data management, and remote accessibility. This review provides actionable insights for researchers and practitioners while identifying future research opportunities to advance scalable and adaptive SHM solutions for large-scale infrastructure.

## 1. Introduction

Structural health monitoring (SHM) is a systematic approach to evaluating the integrity and performance of structures through continuous data acquisition and analysis using a network of sensors. SHM is experiencing significant advancements in sensing technologies. Among the eight axioms of SHM [1], seven emphasize the pivotal role of sensors in designing effective monitoring systems. As a result, substantial investments are made in sensing infrastructure. For instance, SHM systems for wind turbines—which include sensors, data acquisition, and analysis software—typically account for approximately 0.5% to 2% of operational expenditure [2]. The effectiveness of these systems depends heavily on the sensor technologies employed for damage assessment and data collection. Given the rapid evolution of both sensors and analytical techniques, it is essential to continuously refine the underlying methodologies by incorporating new advancements and establishing comprehensive evaluation criteria.

To anchor the review in current research, we begin by assessing recent reviews concerning SHM sensor technologies. Notable contributions include Schubel et al. [3], who explored Fiber Bragg Gratings (FBG) for wind turbine rotor blades, discussing their advantages and limitations. Tchakoua et al. [4] reviewed methods for monitoring and diagnosing wind turbine conditions, focusing on enhancing energy efficiency and addressing mechanical failures. Li et al. [5] focused on damage detection in wind turbine blades using fiber optic and piezoelectric sensors, and Webb et al. [6] highlighted the role of communication and sensing technologies in understanding structural integrity. Other significant studies include Beganovic et al. [7], Ying Du et al. [8], and Burgos et al. [9], who all emphasized the critical role of SHM in improving system reliability and performance. Additionally, Kot et al. [10] discussed Non-Destructive Testing (NDT) techniques in civil engineering, and Laflamme1 et al. [11] reviewed innovative measurement technologies for large structures. A recent contribution was made by Hassani et al. [12], who reviewed sensor technology advances in NDT and SHM of civil structures.

A comprehensive review by Sivasuriyan et al. [13], published in 2024, detailed the integration of various sensor technologies such as piezoelectric, fiber optic, and MEMS devices within SHM systems. These sensors are vital for acquiring accurate data on parameters like force, displacement, and temperature, which are essential for the timely prediction and prevention of structural failures. The review underscores the shift from traditional manual inspections to automated monitoring systems that offer higher precision and reliability. Autonomous sensor technologies represent a significant trend in SHM, particularly in their application to predictive maintenance and structural health assessments. These systems utilize AI to enhance data processing and interpretation, allowing for more proactive maintenance strategies. The integration of the Internet of Things (IoT) in SHM has been identified as a crucial advancement, facilitating the development of sensor networks that enhance the scope and effectiveness of monitoring practices. These networks enable seamless data transmission and processing across vast infrastructures, leading to enhanced monitoring capabilities and more informed decision-making in real-time structural assessments [14]. Optical fiber sensors are noted for their high sensitivity and capability for distributed sensing, making them ideal for large-scale applications in SHM. These sensors provide continuous, real-time data on structural changes, offering vital insights into the health status of structures. The precision and durability of optical fiber sensors make them particularly suitable for challenging environments where traditional sensors might fail [15]. Despite the technological advances, several challenges persist in the field of SHM. These include the integration of sensor technologies with existing infrastructures, the durability of sensors under adverse environmental conditions, and the refinement of data analysis methods to predict structural failures more accurately. Addressing these challenges is crucial for the advancement of SHM and ensuring the long-term reliability and safety of structures [16].

Even though these fields have been extensively researched, there is a significant research gap: while many review articles investigate individual sensing technologies, comprehensive comparative analyses are notably absent. Such an absence arises because direct comparisons across methods are often deemed impossible due to the specificity and tailored nature of each technology. This work addresses the gap by introducing a novel approach to evaluate and compare these diverse technologies through a transparent and structured framework. We propose five main evaluation criteria, further refined with subcriteria, to facilitate a meaningful performance analysis. Although direct comparisons between methods remain infeasible, individual scores are assigned based on grounded literature analyses to provide a robust, data-driven assessment.

This article presents a novel contribution by establishing five comprehensive evaluation criteria to compare the performance of seven state-of-the-art sensor technologies in SHM. These technologies include InfraRed Thermography (IRT), electrochemical sensing, strain measurement, ultrasonic testing, Visual Inspection (VI), vibration and acoustical response. Our criteria are divided into two major categories: deployability, comprising SHM suitability and hardware requirements, and intelligent damage assessment, covering signal characteristics, feature sensitivity, and Digital Twin (DT) simulation. Through this structured framework, one ensures that the scoring process remains objective, emphasizing transparency and traceability. Subcriteria are introduced to provide granular insights, helping researchers and practitioners understand performance nuances without implying a strict ranking hierarchy.

Throughout this analysis, it is emphasized that each technology’s performance is highly context-specific, making cross-comparisons inherently challenging. Nevertheless, the proposed scoring approach helps highlight key strengths, limitations, and trade-offs, offering valuable insights for SHM system design. Furthermore, we provide a future outlook on next-generation sensing technologies, including the integration of self-sensing structures, IoT frameworks, unmanned aerial vehicles (UAVs), and data fusion techniques. These advancements promise to address several inherent limitations of classical SHM technologies by improving scalability, remote accessibility, and the ability to handle large, complex data sets. Enhanced integration with DTs will enable more robust and adaptive monitoring systems, reducing the impact of environmental noise, improving early damage detection, and facilitating real-time predictive maintenance.

The work is structured as follows: Section 2 defines the five evaluation criteria for SHM sensor technologies, covering both deployability and intelligent damage assessment. Section 4.5 reviews the seven state-of-the-art sensor technologies, examining their performance under these criteria. Section 4 and Section 5 present a comparative analysis using visual tools such as ranking tables and heatmaps to highlight key trade-offs. Section 6 explores future advancements in SHM, including IoT, UAVs, and data fusion. Section 7 summarizes the article’s recommendations and presents future research perspectives. A list of abbreviations used throughout this paper is provided in Table 1, ensuring clarity and consistency in terminology.

## 2. Performance Criteria for SHM Sensing Techniques

**Figure 1:**
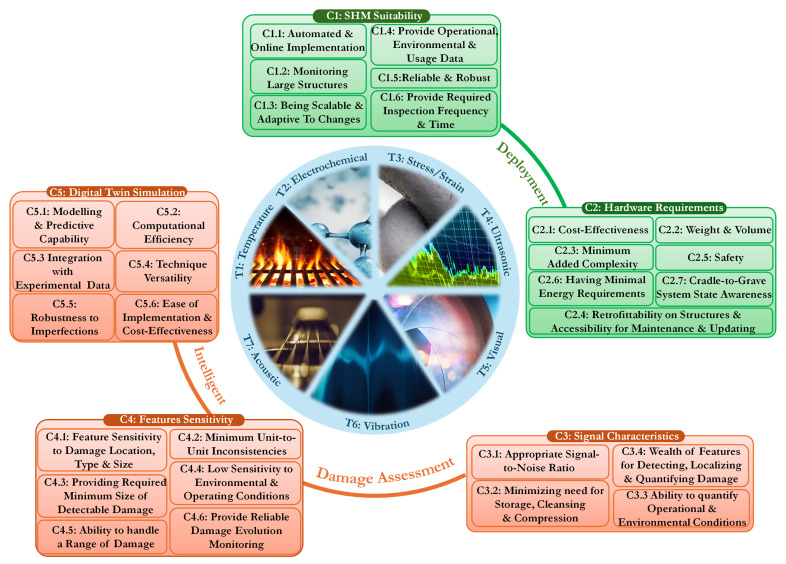
Summary of the performance criteria for the evaluation of SHM systems.

We begin by establishing and describing a set of performance criteria for assessing the suitability of structural sensing technologies for health state diagnosis and prognosis. These criteria are aligned with both the five levels of intelligent damage assessment [17] and practical implementation considerations. The first two criteria address deployment, focusing on suitability and hardware requirements, while the remaining three criteria pertain to intelligent damage assessment, encompassing all stages from damage detection to remaining life estimation. Figure 1 illustrates these criteria and their key highlights.

**(C1) Criterion 1—SHM Suitability:** Structural monitoring aims to enable *(C1.1) Online and Automated Damage Detection and Identification*, distinguishing it from traditional NDT, which is typically performed with the structure taken out of service. An effective SHM system must incorporate sensors able to *(C1.2) Monitor Large Structural Geometries* using minimal equipment. This ensures efficient coverage of expansive infrastructure, unlike conventional techniques that provide localized damage information but require extensive scanning of the investigated structure [18]. Another critical requirement is *(C1.3) Adaptability to a Variety of Operational Evaluation Needs*, ensuring that the system remains effective across different structural components, damage types, and environmental conditions [19]. Furthermore, an optimal SHM system should exhibit the ability to *(C1.4) Provide Operational, Environmental, and Usage Data* at minimum extra cost and weight. This means that parameters such as temperature fluctuations, load cycles, and other operational factors should be captured without imposing excessive additional equipment or energy demands.

The *(C1.5) Reliability of the Sensing System* is a key consideration, meaning that the sensors should have a failure rate lower than that of the structure being monitored. Additionally, robustness is necessary to ensure that the system can still detect damage even if a subset of the sensors fails. To support this, a certain level of sensing redundancy is required, where backup or excess sensors enhance failure tolerance and maintain functionality under potential malfunctions [20]. Finally, *(C1.6) Inspection Frequency and Maximum Inspection Time* play a crucial role in determining the effectiveness of an SHM approach. Higher inspection frequencies allow for more real-time monitoring, while shorter inspection times improve efficiency. These considerations become especially important for self-energized systems, where power availability constraints necessitate strategic balancing between sensing intervals and system longevity.

**(C2) Criterion 2—Hardware Requirements:** Advances in hardware components, such as signal generation, amplification, acquisition, sensing devices, and energy transmission, have significantly driven the field forward. However, the *(C2.1) Cost of SHM Systems* remains a crucial factor, as their expenses must be justifiable compared with the costs of reactive and preventive maintenance to ensure economic viability. Another critical aspect is *(C2.2) Weight and Volume*, particularly in transportation sectors such as aerospace, where additional mass can significantly impact performance and fuel efficiency. To be feasible, SHM systems must be designed with lightweight and compact hardware, ensuring they integrate seamlessly with the structure without compromising efficiency. Closely related to this is *(C2.3) Added Complexity*, which arises due to extensive data acquisition and real-time processing requirements. Many active sensing techniques also necessitate high-voltage amplifiers and signal generators, increasing system complexity and further influencing design considerations [21]. Additionally, complexity often correlates with increased wiring requirements, where extensive cabling for power and data transmission adds to both weight and cost.

To mitigate the challenges of cabling, wireless solutions are often preferred, reducing the structural burden and improving installation feasibility. At the same time, sensing redundancy must be factored in to ensure that failure tolerance is maintained. SHM systems should be flexible enough to support both *(C2.4) Retrofit Applications and Integration* at the manufacturing stage, making hardware accessible for maintenance and upgrades when required. Ensuring *(C2.5) Safety* is paramount, meaning that the SHM system should pose no direct risk to users or operations even in the event of failure. This includes considerations such as the placement of high-voltage components and the safe use of chemical elements within the system. Additionally, *(C2.6) Minimal Energy Requirements* are a desirable feature, allowing monitoring systems to move toward energy autonomy and reducing dependency on external power sources while maintaining long-term operational reliability. Finally, *(C2.7) Cradle-to-Grave System State Awareness* is an essential consideration, ensuring that the system provides continuous monitoring throughout the entire lifecycle of the structure. This means that sensors should not only detect initial structural integrity but also track long-term degradation, damage progression, and repair effectiveness, enabling lifetime predictive maintenance.

**(C3) Criterion 3—Signal characteristics:** Clear and meaningful measurements are fundamental to the effectiveness of any sensing approach, with *(C3.1) Signal-to-Noise Ratio (SNR)* being a critical factor at all levels of assessment. A high SNR ensures that meaningful signals are distinguishable from background noise, improving detection accuracy. However, SNR is influenced not only by sensor and equipment quality but also by operational and environmental conditions, which may degrade signal strength or amplify noise. An optimal sensing system should also *(C3.2) Minimize the Need for Data Storage, Cleansing, and Compression* [22]. Efficient data cleansing involves automatically identifying reliable data for assessment, detecting sensor failures and outliers, and reducing dependence on expert interpretation. Processes such as resampling and filtering contribute to this cleansing effort. Additionally, data compression is important for long-term monitoring, as reducing the dimensionality of acquired data enhances system efficiency and minimizes hardware requirements.

The *(C3.3) Ability to Quantify Operational and Environmental Conditions* is another key requirement, ensuring that structural variations due to external factors such as temperature, humidity, and loading conditions are accounted for. This capability allows for proper data normalization, preventing benign changes from being misinterpreted as damage and thereby reducing the risk of false alarms (Type I errors). Finally, an effective SHM system must possess a *(C3.4) Wealth of Features for Detecting, Localizing, and Quantifying Damage* [23]. A damage feature is any quantity extracted from a signal waveform or structural response that provides indications of structural integrity and deterioration. Ideally, these features should be low-dimensional, computationally efficient, and highly sensitive to damage, minimizing the need for extensive post-processing. Feature extraction may also involve sensor fusion, where data from multiple sensors are combined to enhance reliability and maintain monitoring accuracy even in the presence of sensor failures.

**Figure 2:**
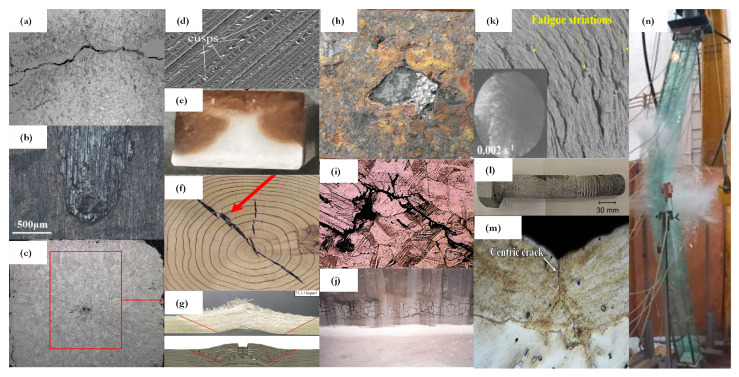
Types of damage and structural conditions detected in large structures. (**a**) surface-level fracture [28], (**b**) erosion [29], (**c**) void formation [30], (**d**) delamination [31], (**e**) thermal shocks [32], (**f**) internal cracking [33], (**g**) impact [34], (**h**) pitted corrosion [35], (**i**) stress corrosion cracking [36], (**j**) hydration swelling [37], (**k**) fatigue [38], (**l**) bolt loosening [39], (**m**) weld join failure [40], (**n**) buckling [41]. Reprinted with permission.

**(C4) Criterion 4—Features Sensitivity:** Having high-quality signals with numerous features is meaningless if those features are insensitive to key damage characteristics. A technique must reliably distinguish between healthy and defective states with a statistically significant certainty, a primary focus of the SHM community [24]. The smallest detectable damage size is a critical threshold [25], influencing the selection of a sensing technique, especially in low-damage-tolerance frameworks such as aerospace and nuclear components. Good detectability alone does not guarantee comprehensive damage assessment. Higher-level damage analysis requires the *(C4.1) High Sensitivity of Extracted Features to Damage Location, Type, and Size*, which is essential for effective diagnosis. An ideal system should not only detect damage but also accurately identify its location, nature, and severity. This is crucial for diagnostic and prognostic decision-making, particularly in complex structural systems where precise damage characterization improves maintenance planning. Damage location, considered Level 2 in damage detection performance, is typically the first information sought after confirming existence (Level 1), guiding detailed inspections and repairs. Classification identifies the type of damage, ranging from small cracks to complex intralaminar microdamage in composites. A selection of prevalent damage types in large structures is illustrated in Figure 2.

Another essential factor is *(C4.2) Minimum Unit-to-Unit Inconsistencies*, ensuring that sensor placement inaccuracies and manufacturing variations do not introduce significant discrepancies in measurements [26]. Variability between units can reduce the reliability of SHM systems, particularly in large-scale implementations where uniform sensor performance is necessary for consistent monitoring. The *(C4.3) Providing the Required Minimum Size of Detectable Damage* is another crucial factor. Many low-damage-tolerance structures require early-stage defect detection to prevent catastrophic failure, significantly impacting preventive maintenance strategies. While sensitivity to damage is crucial, the *(C4.4) Low Sensitivity to Environmental and Operating Conditions* is equally important [27]. SHM systems must ensure that benign variations, such as temperature, pressure, and humidity changes, do not interfere with damage detection accuracy. Engineers typically prefer minimal environmental sensitivity unless these variables are necessary for damage feature extraction.

An effective SHM system should also have a *(C4.5) Sufficient Dynamic Range for the Features to Accommodate a Range of Damage Sizes*. This ensures that the system remains reliable across different defect magnitudes, from microscopic cracks to large-scale failures. A broader dynamic range enhances scalability, making the system versatile across multiple structural applications. Finally, combining fine sensitivity with a broad dynamic range enables *(C4.6) Providing Reliable Damage Evolution Monitoring*, which is a key component of damage prognosis [42,43]. The ability to track damage initiation, propagation, and progression over time is fundamental to predictive maintenance strategies, allowing for informed decision-making regarding structural integrity and longevity. These combined requirements underscore the importance of sensitivity in SHM systems. Beyond detection, features must reliably assess the extent and geometry of all detected damage, ensuring robust monitoring for both immediate diagnostic needs and long-term structural health management.

**(C5) Criterion 5—Digital Twin Simulation:** Multiphysics simulations and data analytics are fundamental to DT technology, enabling continuous refinement of structural health assessments using real-time operational data. This evolution integrates modeling, parameterization, model identification, and SHM, facilitated by advancements in computational algorithms, large-scale optimization, affordable sensors, and wireless data transmission technologies such as IoT and 5G. A well-functioning DT should exhibit *(C5.1) High Modeling Accuracy and Predictive Capability*, meaning that its virtual model must faithfully replicate the physical system under various loading conditions and operational states. This requires the integration of physics-based models, statistical methods, and AI-driven learning techniques to reconstruct real-world scenarios from field measurements, even when simplified computational models are used [44]. To be practical in SHM applications, a DT must offer *(C5.2) Computational Efficiency and Scalability*. This means that model updating and simulations should be computationally feasible for large and complex structures. Advances in high-performance computing (HPC), parallel processing, and surrogate modeling have significantly improved the scalability of DT simulations, making real-time updates more achievable.

Another essential requirement is the *(C5.3) Integration with Experimental and SHM Data*, ensuring that FE models and other simulations remain continuously refined with incoming sensor data [45]. This real-time data assimilation allows SHM models to capture structural changes accurately and improve predictions about damage progression and system health. For maximum usability, a DT should demonstrate *(C5.4) Versatility Across SHM Techniques*. This means that it should support a broad range of sensing technologies, including ultrasonics, strain sensing, vibration analysis, and AI-based methods. The ability to interface with diverse SHM approaches enhances the adaptability and cross-validation of damage detection strategies.

The robustness to *(C5.5) Real-World Imperfections* is another critical consideration, as sensor inaccuracies, environmental disturbances, and incomplete data can degrade the performance of a DT. The system should be resilient to uncertainties, employing statistical learning, sensor fusion, and data-driven correction mechanisms to ensure reliable damage assessments despite imperfect inputs. Finally, a DT must be *(C5.6) Designed for Ease of Implementation and Cost-Effectiveness*. While high-fidelity modeling is valuable, practical adoption of SHM-driven DTs depends on affordable sensor integration, seamless data transmission, and accessible computational resources. Cost-effective deployment ensures that asset management strategies remain economically viable while still benefiting from advanced predictive insights.

## 3. A Summary of the State-of-the-Art SHM Sensing Technologies

**Figure 3:**
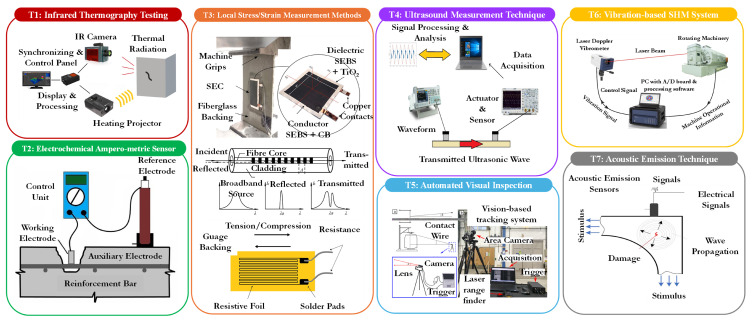
The established sensing methods utilized in SHM systems: (T1) Temperature sensing methods, (T2) Electrical and electrochemical sensing methods, (T3) Local strain measurement approaches (adapted and reprinted from Refs. [53,54] with permission), (T4) Ultrasonic testing, (T5) Automated visual inspection technique (reprinted from Ref. [55] with permission), (T6) Vibration response methods, and (T7) Acoustic response methods (reprinted from Ref. [8] with permission).

This section delves into several established sensing methodologies that have proven instrumental in detecting early signs of degradation, predicting structural failures, and ensuring overall safety. The techniques covered include temperature sensing methods, electrical and electrochemical sensing methods, local strain measurement approaches, ultrasonic testing, automated visual inspection techniques, vibration response methods, and acoustic response methods (see Figure 3). Each of these methodologies offers unique insights into the health of a structure, utilizing different physical properties and technological advancements to monitor structural conditions effectively.

**(T1) Temperature sensing methods (IRT):** Temperature-based SHM utilizes temperature fluctuations as primary indicators of structural integrity for long-span evaluations. Due to significant daily and seasonal temperature variations, these fluctuations cause considerable strains and displacements in large structures, making this approach crucial for long-term structural assessments [46]. Infrared technologies, established since the early 19th century, have proven effective in a wide array of applications, especially in NDT and SHM of civil structures [47,48,49,50,51]. This approach involves monitoring temperature changes on structural surfaces over time using thermographic technologies. IRT splits into passive and active categories, with active thermography further subdivided into techniques such as pulsed thermography, vibrothermography, step heating, and lock-in thermography, with pulsed and lock-in techniques being the most commonly employed in active setups. Both utilize sensitive thermal imaging devices (IR cameras) that can operate in transmission or reflection modes. In transmission mode, the heat source is placed opposite the IR camera across the sample, while in reflection mode, both the heat source and the IR camera are on the same side [52]. The system’s core components include an infrared radiometer for capturing emitted infrared radiation, an energy source for inducing temperature changes, a control panel for adjusting system parameters, and a data processor for creating images that reveal distinct thermal patterns. The fundamental concepts of IRT testing are depicted in Figure 3 (T1).

**(T2) Electrical/electrochemical sensing methods:** Electrical techniques apply electricity to an element to assess various electrical properties, providing insights into the material’s structure. Electrochemical methods measure potentials or responses, offering critical data on material interactions [12]. Key electrical and electrochemical techniques in SHM include the following [56,57]:

- Corrosion current: measures the electrical current from electrochemical reactions on a structure’s surface to monitor and mitigate corrosion.
- Electrical conductivity: assesses a material’s ability to conduct electricity, which is crucial for evaluating conductive materials’ integrity.
- Corrosion half-cell potential: gauges electrochemical potential differences to identify corrosion-prone areas.
- Capacitive/resistive humidity sensors: detects changes in humidity through variations in electrical capacitance or resistance, indicating potential structural damage.
- Alternating current field measurement: uses alternating electromagnetic fields to detect surface defects in conductive materials, applicable in various industries.

Electrochemical sensors, categorized into potentiometric, amperometric, and conductometric types, use electrodes to interact with a chemically selective layer for real-time composition analysis. Potentiometric sensors measure potential differences to determine composition, amperometric sensors measure current flow through oxidation or reduction, and conductometric sensors assess material conductivity [12]. A typical amperometric sensor, illustrated in Figure 3 (T2), includes an auxiliary electrode, a counter electrode, and a reference electrode. This type of sensor identifies the flow of current by gauging the oxidation or reduction in an electroactive element between a working electrode and a reference electrode.

**(T3) Local strain measurement:** The method evaluates structural damage by examining changes in strain distributions at critical locations arising from the realignment of load pathways, which is crucial for assessing structural integrity [58]. Strain measurement is typically indirect [3], employing two main types of sensors: conventional electrical strain gauges and modern fiber optics. Electrical strain gauges, particularly resistance sensors, are prevalent due to their versatility, detecting changes in the resistance of a wire as it stretches and correlates directly with displacement (Figure 3 (T3)).

The Soft Elastomeric Capacitor (SEC), among capacitive sensors, is notable for measuring strain in large structures. It utilizes a thin film sensor design, converting structural strains into capacitance variations, featuring a styrene–ethylene–butylene–styrene matrix with titania for increased durability and permittivity and carbon black-doped layers as conductors [59,60] (Figure 3 (T3)). In fiber optics, their adoption of SHM, particularly in wind turbine blades, is increasing [8]. Simple systems, such as intensity-based fiber optic sensors, measure the intensity of light reflected back to a detector, which varies with alignment changes. More advanced systems like the Fabry–Pérot interferometers and FBG sensors analyze light interference patterns and wavelength shifts in reflected light due to structural deformations to determine strain [15,61] (see Figure 3 (T3)).

**(T4) Ultrasonic testing:** The technique for inspecting structural integrity using ultrasound involves transmitting ultrasonic waves through a structure to detect and evaluate damage and predict mechanical property changes [4,10]. This approach uses transducers to emit pulses ranging from 0.2 to 15 MHz, with higher frequencies enhancing resolution and precision [12]. These waves, when interacting with different materials, provide insights into the damage’s size and location by reflecting, attenuating, and transmitting differently [62]. Ultrasound-based non-destructive evaluation employs acoustoelasticity, where elastic wave properties are influenced by mechanical stresses [63,64]. It includes contact methods using piezoelectric transducers with gel couplants and non-contact air-coupled methods using air as the medium [10,65]. Ultrasonic Guided Waves (UGW), particularly Lamb waves in plate-like media, are used for SHM and damage detection due to their low attenuation and high frequency, which are capable of identifying small defects [66]. These systems analyze wave propagation, comparing it with known responses for damage detection via pattern recognition [17,67,68].

Lamb waves, arising from compression and shear wave interactions, are influenced by structural boundaries and damage. Detected by ultrasonic sensors, they convert into electrical signals [69]. Various configurations like pulse-echo, pitch-catch, and phased-array are used for damage detection, with phased-array systems particularly effective in controlling wave direction through timed transducer arrangements [70]. PWAS are highlighted in SHM for their cost-effectiveness and broad-spectrum capabilities, often configured into arrays for active monitoring [71,72].

**(T5) Automated visual inspection techniques:** Automated visual inspection systems utilize advanced video cameras to remotely monitor the health of structures. These camera-based SHM methods, combined with various signal-processing techniques, have been extensively applied in monitoring buildings and bridges [73,74,75,76,77,78,79,80,81,82,83,84,85,86,87,88,89]. These techniques primarily focus on visualizing structural integrity, motions, and quantitative vibration, and displacement measurements [81]. Such methods are integral to broader SHM systems, aiding in determining a structure’s serviceability and maintenance schedule. Particularly for wind turbine blades, vision-based methods provide critical insights into deformation patterns under various loads, as highlighted in numerous studies [90,91,92,93,94,95,96]. T. Jiang et al. introduced a portable vision-based tracking system for non-contact vertical displacement measurements using a novel line-tracking technique that performs well in noisy environments [55]. Meanwhile, K. Jang et al. developed a hybrid image scanning system that combines vision with laser IRT and proposed a CNN-based autonomous concrete crack evaluation algorithm [78].

Motion magnification algorithms, both Lagrangian and Eulerian, amplify minuscule motions in videos at specific frequency bands. A notable advancement is phase-based motion magnification, which enhances nearly imperceptible structural movements using complex-valued image pyramids [97,98,99]. Additionally, remote sensing techniques like LiDAR and photogrammetry have significantly advanced, providing accurate 3D structural information through laser pulses integrated with GPS or inertial measurement systems and processing images to measure distances and create geometric representations [45,100,101,102,103]. The evolution in computer science and optics has fostered machine vision-based methods for long-distance, low-cost damage detection and dynamic identification [104,105,106,107]. Dong and Necati Catbas reviewed the applications of computer vision (CV) in SHM, indicating potential new opportunities, although not all computer vision research can be directly applied to SHM [107]. The use of CV and Machine Learning (ML) in automating crack detection and identification in civil infrastructure SHM systems is increasingly pivotal, as demonstrated by Islam and Kim’s autonomous system combining machine vision with deep CNNs for large concrete structures [76].

**(T6) Vibration response methods:** Vibration-based SHM systems monitor abnormal oscillations and deformations, linking them to specific damage types through structural dynamics such as frequency response and modal parameters [108]. These systems utilize various sensors—displacement sensors, velocity sensors, and accelerometers—to cover low, medium, and high-frequency ranges, respectively [4,109,110]. Accelerometers, due to their broad frequency spectrum, are widely used across industries [8]. Laser Doppler Vibrometers (LDVs) enable contactless vibration measurements by analyzing backscattered light from a structure’s surface [111,112], while ground-based radar systems are effective for monitoring large infrastructures like bridges [113,114,115].

Vibration response techniques are broadly classified into model-based and non-model-based approaches [116]. Model-based methods rely on analytical models for damage assessment, whereas non-model-based methods compare conditions between damaged and undamaged states. Piezoelectric accelerometers, widely applied in civil and aerospace industries, are valued for their durability and broad operating range, although they cannot measure static accelerations [12]. Erduran et al. [117] highlighted promising advancements in damage detection precision for bridge monitoring, though further validation is needed. Microelectromechanical Systems (MEMS) accelerometers, known for their compact size and affordability, have become prevalent in electronics. These devices employ various mechanisms, including capacitive and ohmic switches, though long-term degradation may affect performance. While MEMS accelerometers offer advantages in cost and energy efficiency, they may provide lower precision compared with piezoelectric types in SHM applications [118].

**(T7) Acoustic response methods (AE):** Acoustic techniques have been used for SHM since the 1970s [119]. Here, the source of the generated transient acoustic waves is damage initiation, crack propagation, or local plastic deformation. The transduction of this released energy to the acquired electric signals is performed by the acoustic transducers attached on or set toward a certain point of the structure [120]. The working principle of Acoustical Emission (AE) is very straightforward and is depicted in Figure 3 (T7). The AE sensors are the central component of the AE system, converting waves into electrical signals. Non-integral sensors require an amplifier before transferring the signal to the AE instrument, whereas integral sensors with embedded amplifiers can transmit the signal directly to the instrument. A data acquisition system is then used to acquire the signal. There are a number of factors that affect the signal waveform, including the path from the source to the sensor, the characteristics of the sensor, and the measurement system. AE features include energy, amplitude, duration, hit, and rise time (the delay between initiation and highest peak) [10].

**Figure 4:**
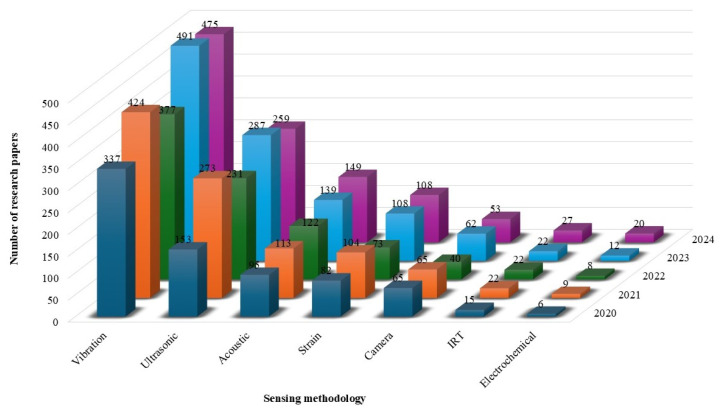
The recent research papers on the application of the established SHM sensing methods over the past five years. The x-axis categorizes the different sensing techniques, while the y-axis represents the number of research papers published. The bars are color-coded to differentiate publications from 2020 to 2024, showing the progression of research in each method. The data come from keyword searches in Scopus.

The bar chart in Figure 4 presents the number of review articles published over the past five years (2020–2024) concerning the seven different sensing methodologies. Vibration-based SHM and ultrasonic methods are the most researched techniques, respectively. Acoustic, strain-based, and camera-based monitoring have moderate research activity, while IRT and electrochemical sensing remain niche areas with limited publications. The data highlight a growing trend in camera-based and vibration response methods while other sensing techniques continue to be explored at a lower scale. The data come from keyword searches in Scopus. The extracted data are confined to journal articles, books, and conference proceeding publications specifically relevant to a structural health monitoring case study.

Despite the large volume of publications, no existing studies have systematically compared these different sensing methods using specifically defined evaluation criteria. In the following section, we address this gap by providing a structured framework to facilitate a performance-based analysis across diverse SHM technologies.

## 4. A Comparison of SHM Sensing Technologies Based on Performance Criteria

In this section, we provide a detailed comparative analysis of the seven SHM sensing technologies discussed in Section 4.5, focusing on their attributes, strengths, and integration potential within SHM systems. Rather than ranking these technologies—a task rendered impractical due to the highly tailored and context-specific nature of each method—we assign performance scores on a scale of 1 to 5. These scores are based on subcriteria defined in Section 2, allowing for structured and meaningful evaluations without implying superiority among methods.

**Figure 5:**
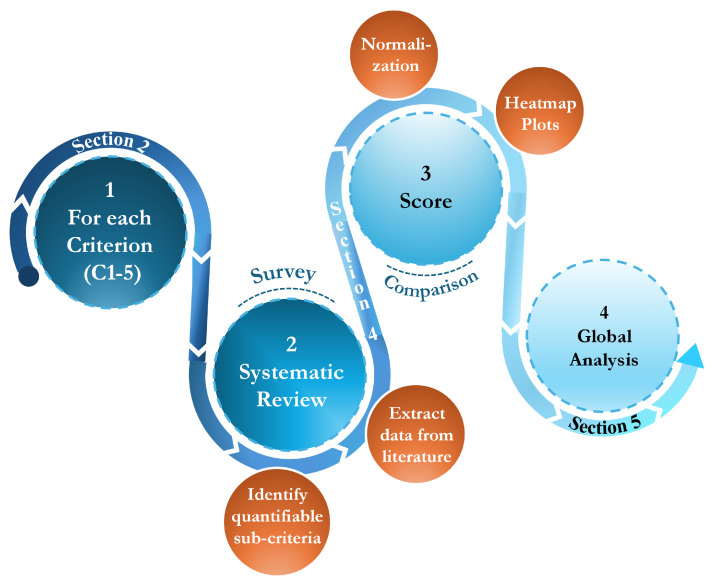
A flowchart describing the comparative analysis of SHM sensing technologies T1–T7 vis-à-vis criteria C1–C5.

This analysis is grounded in a synthesis of state-of-the-art literature and aims to highlight key performance trends across multiple criteria. To aid in visualization and interpretation, heatmaps are used to present the performance scores, fostering informed discussions about the trade-offs and applicability of each technology. These insights are subsequently consolidated in Section 5, where we provide a comprehensive synthesis of findings. An overview of the entire methodology is illustrated in Figure 5.

### 4.1. (C1) Criterion 1: SHM Suitability

**(C1.1) Automated and Online Implementation:** Automated SHM systems offer the potential for continuous, real-time monitoring without operational interruptions. IRT is effectively used for detecting delaminations and defects in concrete structures, which are typically invisible to the naked eye. This method takes advantage of rapid operational efficiency, utilizing non-intrusive, contactless thermographic technology for real-time monitoring [46,121]. Electrochemical sensors deliver spatially distributed measurements for a comprehensive structural evaluation and real-time monitoring of electrical resistance, aiding in the timely detection of structural changes and facilitating prompt maintenance [11]. While strain gauges are effective for online and automated monitoring, their performance in demanding environments, like those experienced by wind turbine blades, is surpassed by FBGs, making them a preferable choice in such applications [122]. AE techniques are valuable for detecting crack propagation in metallic structures, sensitive enough to detect subtle acoustic signals emitted from crack formations [123,124]. By adding wireless communication to the AE technique, it’s possible to create a strong and dependable in situ SHM system [125,126]. PWAS in phased array configurations is the low-cost, commercially available, and most reliable ultrasonic technology for SHM, enabling online, automated, and on-site monitoring of structural health [71,127,128,129]. MEMS accelerometers offer continuous and steady measurement capabilities. Their cost-effectiveness and low power usage enable the establishment of a monitoring infrastructure that provides continuous and real-time data [108,109]. The machine vision system can capture images at a rate of 5 frames per second, and the entire image processing can take 200 ms per frame. The online application of CV-based SHM can face challenges when the system requires analysis at high frame rates, as demonstrated by industrial cameras operating up to 5000 frames per second in specific monitoring scenarios [45,107].

**(C1.2) Monitoring Large Structures:** Techniques such as IRT and Visual Inspection are particularly advantageous for large-scale monitoring. They cover extensive areas efficiently and are less dependent on extensive hardware setups [46,81]. Vibration-based methods have seen significant advancements with MEMS accelerometers, which are now capable of detecting modal frequencies with high accuracies, reported in [118] within 1.6% error, making them suitable for monitoring bridges, wind turbines, and buildings [12]. Covering large structures with AE sensors means spreading them widely, which increases the number of sensors needed as well as the cost and weight of the system [5]. Ultrasonic phased arrays, particularly those using PWAS, enable inspections over large areas from single points, dramatically reducing the need for extensive sensor networks [130]. However, strain-based methods, such as those used in wind turbine blade monitoring and electrochemical sensors for corrosion monitoring, still require extensive sensor networks to achieve comprehensive coverage, significantly increasing both cost and system complexity [3,58].

**(C1.3) Being Scalable and Adaptive to Changes:** Scalability is crucial for deploying SHM technologies across various structures. IRT and vibration sensors are easily scalable due to their minimal setup requirements [12,46]. AE and ultrasonic methods require sensor arrays but offer adaptability through advanced signal processing techniques [5,129]. Ultrasonic phased arrays using PWAS can inspect in a range of 0.2–1 m^2^ from a single location, reducing sensor count [69]. Strain-based FBG sensors enable real-time damage tracking with a micro-strain resolution of 1*με* but require dense sensor networks, increasing installation complexity and resulting in changeable scalability [8,122]. Electrochemical sensors measure corrosion rates in steel with an accuracy of ±5% but require recalibration every 6 months to maintain precision, affecting long-term scalability [131,132].

**(C1.4) Providing Operational, Environmental, and Usage Data:** Effective SHM systems should provide insights beyond damage detection. Strain-based methods, particularly FBG sensors, capture structural deformation and temperature variations simultaneously [8,133]. IRT assesses heat distribution patterns, which is useful for detecting environmental effects on structural performance [134,135]. Vibration-based systems provide real-time tracking of dynamic responses, with sampling rates up to 10 kHz and the ability to detect modal frequency shifts as small as 0.2 Hz, ensuring precise assessment of operational loads [109]. However, electrochemical sensors primarily focus on corrosion-related metrics, with polarization resistance measurements in the range of 10^2^–10^6^Ω·cm^2^, limiting their ability to capture broader environmental variations [56,131]. Ultrasound and acoustic signals are capable of conveying detailed information about operational and environmental conditions. However, extracting these data accurately necessitates the application of sophisticated, physics-based signal processing and data analysis techniques.

**(C1.5) Being Reliable and Robust:** Robustness under varying environmental conditions is a key requirement for SHM. Strain-based FBG sensors and accelerometers maintain high reliability even in harsh conditions, with FBG sensors exhibiting stability over temperature variations of −40 °C to 80 °C while maintaining a wavelength sensitivity of about 10 pm/°C [136]. MEMS accelerometers, tested in real-world applications, showed a noise floor of 25 μg/Hz and a power consumption reduction of up to 80% in low-power modes, making them suitable for long-term monitoring [118]. Ultrasonic and AE techniques provide fault-tolerant monitoring through sensor redundancy [126,137]. However, IRT is highly sensitive to environmental factors such as rain, dust, and temperature fluctuations, with studies indicating that steel rope failure detection accuracy decreases by 43.01% when the ambient temperature increases by 10 °C [134]. Electrochemical sensors degrade over time, with experimental data showing that chloride-induced corrosion increases polarization resistance variability by up to 37% in reinforced concrete structures, requiring frequent calibration and maintenance [131,132].

**(C1.6) Providing Required Inspection Frequency and Time:** SHM systems must balance accuracy and efficiency. IRT allows rapid, real-time inspections, with thermal cameras operating at frame rates of up to 60 Hz and capable of detecting small temperature differentials, making them highly time-efficient for large structures [136]. Vibration-based methods provide continuous monitoring with minimal downtime. In [138], the MEMS-based system for the SHM of rotating machinery exhibited satisfactory accuracy in assessing both frequency and acceleration amplitude, consistently achieving a mean absolute percentage error (MAPE) below 5%. Ultrasonic arrays, while effective and capable of high-frequency inspection, require controlled conditions and careful setup, with inspections taking an average of 15–30 min per scan, depending on the material and defect size [129,139]. Electrochemical and strain-based methods provide long-term monitoring but require periodic recalibration, with electrochemical sensors needing recalibration every 4–6 months and strain-based sensors showing drift rates of 0.01–0.02% per day, affecting inspection efficiency [8,131].

**Figure 6:**
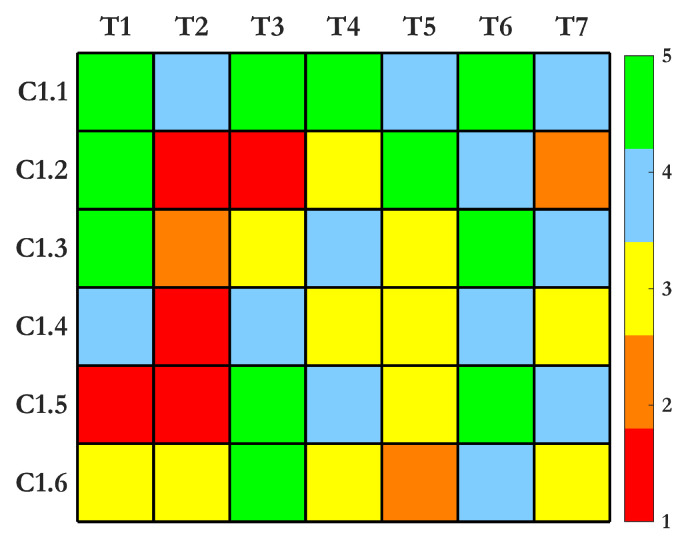
Subcriterion heatmap scores based on SHM suitability.

The heatmap for Criterion 1 in Figure 6 demonstrates how SHM techniques vary in their effectiveness across subcriteria. Techniques such as UGW and strain-based sensors excel in multiple categories due to their scalability, sensitivity, and reliability under diverse conditions. These methods are particularly robust and capable of providing comprehensive data regarding structural deformation, operational loads, and defect propagation. Conversely, IRT and electrochemical sensors show limitations, particularly in terms of scalability and sensitivity to environmental factors, as their performance can degrade under fluctuating conditions. IRT is strong in rapid and large-area inspections but struggles with environmental robustness. AE and machine vision offer excellent online implementation and sensitivity to crack detection but require complex signal processing and noise management to maintain accuracy. MEMS accelerometers display balanced performance, excelling in real-time operational monitoring while being both cost-effective and reliable under harsh environments. Overall, the heatmap indicates that the most versatile SHM methods are those that can adapt to both large-scale monitoring needs and varying environmental influences. The advantages and drawbacks of each method are summarized in Table 2.

### 4.2. (C2) Criterion 2: Hardware Requirements

**(C2.1) Cost-Effectiveness:** The global SHM market is expected to grow from USD 1.7 billion in 2021 to USD 3.8 billion by 2027, fueled by advancements in affordable technologies such as thermal-based systems [12]. Electrochemical sensors, including electrical resistivity methods, offer automated, low-cost solutions for monitoring in harsh environments like tidal zones [131,150]. Fiber optic sensors and SECs provide scalable options with minimal hardware, achieving resolutions of 25 *μϵ* with standard data acquisition tools [151]. Ultrasonic techniques with piezoelectric wafer sensors cover large areas efficiently [141,152]. Camera-based systems eliminate extensive sensor networks, enabling long-range inspections of up to 175 m [153]. However, large installations like the Tsing Ma Suspension Bridge system cost USD 8 million, highlighting the need for economical wireless alternatives [154]. MEMS accelerometers reduce costs significantly compared with piezoelectric sensors [118], while AE monitoring cuts maintenance costs by up to 35% and improves turbine lifespan by reducing downtime [4].

**(C2.2) Low Weight and Volume:** Lightweight infrared sensors and UAV-mounted cameras enable SHM deployment in remote areas without imposing significant structural load [51,135]. MEMS electrochemical sensors are compact, reducing manual inspection requirements in areas like bridge tidal zones [131]. FBG sensors add minimal mass and provide sub-millimeter resolution, which is ideal for precision strain monitoring [155,156]. Ultra-thin piezoelectric sensors facilitate large-area ultrasonic monitoring in aerospace applications [152]. Portable cameras, such as the Canon VIXIA HF R42, weigh significantly less, simplifying deployment [107]. MEMS sensors support extensive vibration monitoring with a lightweight footprint [118], while AE sensors, including 28 µm piezoelectric Polyvinylidene fluoride variants [157], add negligible weight and maintain mechanical integrity under fatigue stress [158].

**(C2.3) Minimum Added Complexity:** Non-contact IRT devices simplify installation and eliminate the need for structural modifications [51,135]. Electrochemical systems require sensor calibration but provide long-term, non-destructive corrosion monitoring [131]. Integrating ultrasonic measurement techniques into SHM systems adds significant complexity due to the need to manage multimodal wave behaviors and equipment requirements [159]. Cameras with programmable synchronization, such as the Basler ace-acA2040, enhance real-time displacement tracking [107,160]. Automated modal analysis efficiently handles large datasets, accurately monitoring resonance frequencies [146]. The MEMS sensors reliably detect modal frequencies with satisfactory accuracy in assessing both frequency and acceleration amplitude [118,138]. These sensors do not introduce additional complexity to the SHM system due to their small size and self-contained nature. The embedded AE transducers remain functional up to 90% of failure and remain unaffected by damage in both static and fatigue loading. [158]. These AE techniques show the capability to integrate smoothly into SHM setups without significantly increasing system complexity due to their compatibility with existing monitoring technologies.

**(C2.4) Being Retrofittable on Structures and Accessible for Maintenance and Updating:** Lightweight thermal sensors are easily retrofitted with minimal structural disruption, allowing efficient long-term monitoring [12,135]. Embedded and wireless electrochemical sensors automate corrosion monitoring on existing concrete structures with remote accessibility for updates [131]. Strain gauges and fiber optic sensors require no major alterations and are successfully deployed on critical infrastructure, including bridges and wind turbines [3,155]. LDVs and air-coupled ultrasonic sensors allow flexible installations without coupling agents [141,161]. Cameras can monitor structures from up to 80 m away, improving accessibility for large projects [81]. Wireless sensor networks cut retrofitting costs, as seen in historic sites like Consoli Palace, where DTs continuously update monitoring data [144]. AE sensors reduce installation time significantly and offer adaptable retrofitting for both new and existing structures [157,158,162].

**(C2.5) Safety:** Temperature-based approaches improve safety by reducing human exposure to hazardous areas through non-invasive, remote sensing [51,160]. Electrochemical sensors utilize corrosion-resistant materials and insulation to operate safely under fluctuating environmental conditions [131]. Strain-based systems, including fiber optics, pose no safety risks due to their inert, interference-free properties [156,163]. Ultrasonic and laser-based sensors minimize mechanical risks by operating without physical contact [139,161]. Camera systems enable safe remote inspections [81,160], while vibration monitoring reduces failure risks by identifying structural anomalies early [145]. AE sensors, with low power consumption and no emissions, function reliably under harsh conditions [4,157,158].

**(C2.6) Having Minimal Energy Requirements:** Thermal systems use passive energy sources like sunlight, with infrared sensors and UAVs optimized for extended operation [47,134]. Wireless electrochemical sensors face energy challenges from continuous monitoring, prompting research into RFID-based low-power alternatives [131]. Fiber optic systems require minimal energy, supporting stable long-term monitoring in remote locations [155]. Camera systems powered by low-voltage batteries demonstrate energy efficiency in field applications [160]. MEMS accelerometers reduce power needs by up to 80% in low-power modes [118], while AE sensors are also capable of operating in energy-limited environments, enabling long-term monitoring.

**(C2.7) Cradle-to-Grave System State Awareness:** IRT ensures comprehensive lifecycle monitoring with real-time data collection and low maintenance needs [12,47]. Electrochemical sensors track parameters such as corrosion, pH, and humidity, facilitating preventive maintenance across the structure’s life [56,131]. Integrated SHM systems continuously gather data, as demonstrated in multi-year bridge monitoring, where statistical controls detect sensor anomalies [155]. Ultrasonic systems automate defect detection through guided wave models [139,161]. Cameras, coupled with DTs, enable condition tracking and asset management [143]. Vibration-based systems at Consoli Palace utilize DTs to monitor progressive damage via modal updates [144].

**Figure 7:**
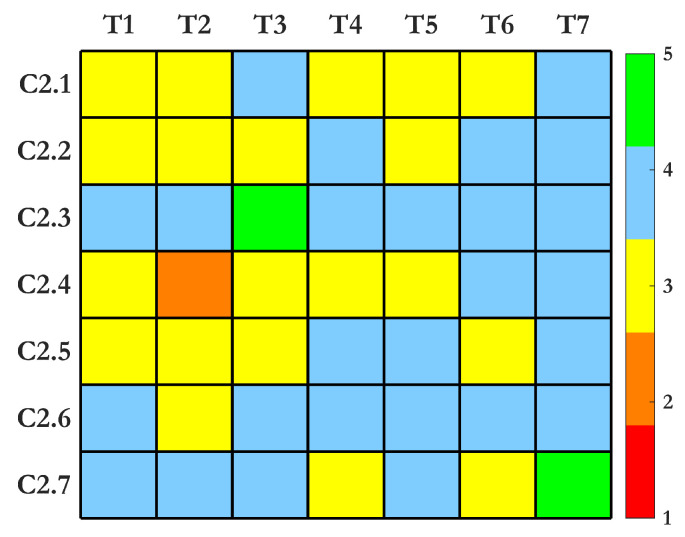
Subcriterion heatmap scores based on hardware requirements.

The heatmap in Figure 7 highlights how SHM techniques perform across cost-effectiveness, system complexity, and lifecycle monitoring. Strain-based (FBG) and AE methods consistently score high across most subcriteria, offering a strong balance of efficiency, scalability, and reliability. Vibration-based systems are also effective, particularly in cost-efficiency and safety, while maintaining low implementation complexity. Ultrasonic and visual techniques score moderately, with visual methods excelling in retrofit applications but requiring higher energy and storage resources. Thermal and electrochemical systems offer competitive performance for long-term lifecycle awareness and ease of implementation, though they require better optimization to minimize environmental sensitivity and energy demands. Overall, these insights emphasize the need to tailor SHM solutions to both technical and budgetary constraints. The advantages and drawbacks of each method are summarized in Table 3.

### 4.3. (C3) Criterion 3: Signal Characteristics

**(C3.1) Having an Appropriate SNR:** Signal clarity varies across sensing techniques, requiring noise mitigation strategies. IRT experiences sufficient SNR drops due to ambient temperature fluctuations, necessitating preprocessing for meaningful thermal extraction [47]. Vibration-based SHM is highly susceptible to environmental noise, with wind-induced fluctuations causing up to 5% deviations and temperature-induced variation up to 10% in natural frequency estimates [19]. MEMS and piezoelectric accelerometers suffer from noise floors that significantly affect low-frequency readings [154]. AE signals are often contaminated with assorted noise factors that degrade the signal clarity and integrity, complicating precise analytical evaluations [167]. Strain-based FBG sensors provide sub-microstrain resolution with minimal susceptibility to environmental interference, offering the highest SNR [163]. The effectiveness of ultrasonic SHM methods is often limited by challenges in noise processing, which impact the signal-to-noise ratio (SNR) and the accuracy of defect estimation [10]. Computer vision-based SHM is susceptible to various noise sources such as electric noise, lens distortion, illumination changes, and environmental factors like wind and vibrations, which introduce measurement uncertainties and affect response accuracy [107]. Among these, strain-based sensing offers the most robust SNR, while IRT and AE require extensive correction for reliable damage detection.

**(C3.2) Minimizing the Requirement for Data Storage, Cleansing, and Compression:** Data storage needs vary significantly across SHM techniques, requiring compression and filtering for efficiency. Infrared thermography (IRT) generates large volumes of image data, necessitating efficient compression and storage management techniques to handle high-resolution measurements effectively [50]. CV-SHM generates very large datasets per bridge inspection, demanding advanced segmentation and reduction techniques [84]. Electrochemical sensors in SHM rely on real-time data acquisition and wireless transmission, necessitating efficient filtering techniques to mitigate environmental interference and optimize data storage and processing [150]. Wavelet transform techniques are widely used in ultrasonic SHM for de-noising and data compression, aiding in efficient signal processing and storage management [164]. Vibration-based SHM leverages frequency domain transformations to reduce data volume and enhance feature extraction, optimizing storage and analysis efficiency [168]. Acoustic Emission (AE) monitoring systems generate large volumes of transient elastic wave data, necessitating efficient filtering and data management techniques to separate meaningful signals from background noise [169]. Strain-based FBG sensors generate compact datasets requiring only periodic logging, making them the least storage-intensive [163]. Strain-based and vibration-based techniques minimize storage demands, while IRT and VI require advanced compression for large-scale feasibility.

**(C3.3) Ability to Quantify Operational and Environmental Conditions:** SHM techniques must distinguish structural damage from operational and environmental influences. Strain-based FBG sensors enable real-time quantification of strain and temperature variations in harsh operational environments, ensuring accurate structural health monitoring [8]. IRT provides thermal mapping but requires emissivity correction and ambient compensation to enhance accuracy in defect detection [134]. VI image quality is highly sensitive to lighting conditions, with illumination changes causing significant misclassification errors [170]. Electrochemical sensors monitor corrosion rates via polarization resistance shifts, but potential variations due to environmental factors require correction [131]. Ultrasonic SHM experiences near-field distortions above 50 °C, demanding stable transducers for accurate readings [164]. Vibration-based SHM sees 10% modal frequency shifts from seasonal temperature variations, requiring baseline compensation [19]. AE technology provides a unique capability to quantify both the operational load and the environmental conditions affecting a structure, thereby enabling effective real-time structural health monitoring [171]. Strain-based and electrochemical methods offer the most robust environmental tracking, while vision-based and ultrasonic techniques require extensive corrections.

**(C3.4) Wealth of Features for Detecting, Localizing, and Quantifying Damage:** The effectiveness of SHM depends on the richness of extracted damage features. Ultrasonic testing provides high-resolution imaging, detecting sub-millimeter defects with guided waves and nonlinear resonance techniques [172]. Acoustic Emission (AE) techniques detect microcracks at low energy thresholds and enable precise defect localization through advanced signal processing methods. [173]. Strain-based sensor arrays, such as SEC networks, enable high-resolution 2D strain mapping, improving structural model refinement through advanced interpolation techniques. [60]. Electrochemical sensors provide rich, multi-parametric data, capturing chloride ingress, corrosion rate, resistivity, and environmental influences in real time [131,150]. Vibration-based techniques provide global structural assessments by detecting modal frequency shifts, which can indicate structural changes, though their sensitivity to localized damage is limited [168]. Infrared thermography (IRT) enables the detection of thermal anomalies and subsurface defects in concrete structures, with the minimum detectable defect size depending on material properties, thermal contrast, and sensor resolution [150]. VI-based Structural Health Monitoring (SHM) techniques offer a wealth of features for detecting, localizing, and quantifying damage by capturing high-resolution displacement and vibration data remotely, enabling detailed structural assessments without direct sensor contact [81]. Ultrasonic and AE methods offer the richest feature sets, while strain and electrochemical methods provide valuable quantitative insights into structural behavior.

**Figure 8:**
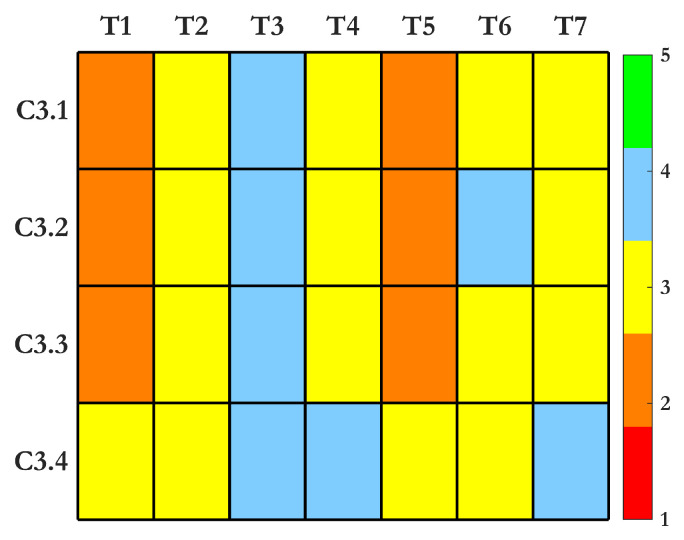
Subcriterion heatmap scores based on signal characteristics.

The heatmap in Figure 8 illustrates the varying performance of SHM techniques under Criterion 3 for feature sensitivity and operational demands. Strain-based (FBG) sensors perform consistently well across all subcriteria, offering a strong SNR, minimal storage needs, robust environmental tracking, and a rich set of damage features. Ultrasonic and AE methods also provide high-quality feature sets and strong performance but require more data storage and noise filtering. Electrochemical techniques offer balanced performance, excelling in tracking environmental conditions, although they demand corrective filtering due to potential variations in readings. Thermal (IRT) and vision-based methods struggle with environmental sensitivity and storage demands, limiting their effectiveness without extensive preprocessing. Vibration-based systems demonstrate efficient data storage and moderate sensitivity but face challenges with noise in dynamic conditions. These results emphasize the importance of selecting techniques that match operational needs and data management capabilities. The advantages and drawbacks of each method are summarized in Table 4.

### 4.4. (C4) Criterion 4: Features Sensitivity

**(C4.1) High Sensitivity of Extracted Features to Damage Location, Type, and Size:** UGW detect 2.77 mm defects in quasi-isotropic composites with 99% confidence, optimizing sensitivity at 54 kHz (A0 mode) and 255 kHz (S0 mode) [72,152]. AE identifies microcracks at 10^−12^ J energy thresholds, localizing AE events in high-frequency bandwidths of 100 kHz–1 MHz [4,7]. Electrochemical methods detect corrosion initiation below −350 mV, and polarization resistance drops to 5 × 10^4^ Ω· cm^−2^, signaling active corrosion [131,132]. IRT detects subsurface defects in composite structures up to 2.5 mm deep with 0.01 °C thermal resolution [51], achieving <4% detection errors [12]. Vibration-based SHM identifies 0.5–2% shifts in natural frequency [146]. Among these, ultrasonic and AE methods provide the highest sensitivity, while IRT and electrochemical techniques excel in early-stage defect detection.

**(C4.2) Minimum Unit-to-Unit Inconsistencies:** FBG sensors achieve ±0.7 μ*ε* repeatability [163], though temperature sensitivity necessitates correction [3]. AE sensors can consistently separate damage events over extended measurement periods ranging from 10 to 100,000 s [12]. Electrochemical techniques exhibit deviations ranging from 0.1 to 1 in resistivity values due to ionic diffusion but stabilize due to probe designs optimized from galvanostatic current techniques in a range of 10 μA–20μA [131]. IRT is affected by emissivity variations, causing ±5% errors [51], while camera-based SHM introduces >5% modal frequency errors due to misalignment [81]. Among these, FBG and AE sensors are the most consistent, while camera, electrochemical, and MEMS sensors require stricter controls.

**(C4.3) Providing the Required Minimum Size of Detectable Damage:** AE identifies defects up to 5 layers deep as well as within a few millimeters in CFRP materials [7], while ultrasonic DTs detect 2.77 mm defects in composites with 99% confidence [72]. Electrochemical sensors register relative depths of concrete carbonation of >1, marking corrosion onset [131]. IRT detects delaminations as shallow as 0.4–0.6 mm [51], though depth sensitivity decreases. FBG sensors resolve strain changes in 1 μ*ε* at 0.1 °C [12], while camera-based DIC methods measure 5 μm crack openings [178]. AI-enhanced spectral analysis refines vibration-based crack detection to 2 mm, though traditional methods require 10 mm damage to affect modal properties [166]. Among these, ultrasonic and AE provide the highest micro-scale sensitivity, while FBG and DIC methods excel in high-resolution strain tracking.

**Figure 9:**
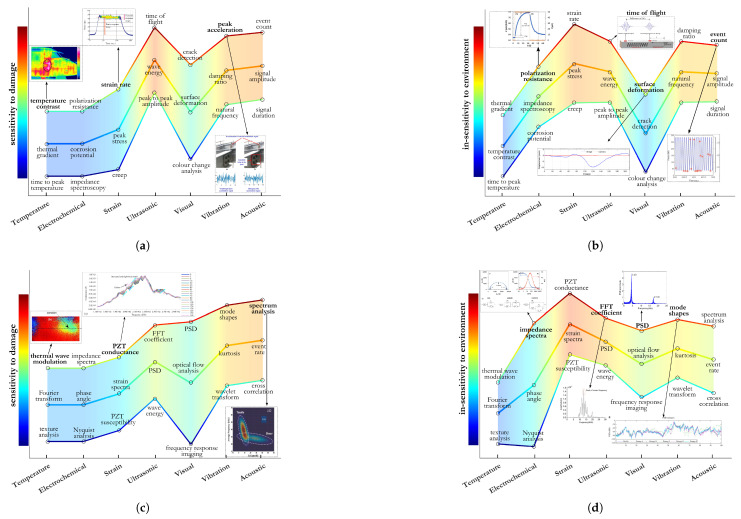
Sensitivity of the most important signal features to damage and environmental factors, according to the literature. All reprinted figures have permission from the original publisher. (**a**) Sensitivity of time domain features to damage: (i) *Temperature Contrast*: insulation failures in aircraft cockpit [183], (ii) *Dynamic Strain*: structural response to wind loads, as measured by FBG1 sensors [184], (iii) *Peak Acceleration*: damaged and undamaged vibration signals measured at the joints [185]. (**b**) Sensitivity of time domain features to environment: (i) *Polarization Resistance*: evolution of potential during charge and discharge [186], (ii) *Time of Flight*: localization of beam damage with actuators and sensors [187], (iii) *Surface Deformation*: camera motion and mid-span vertical displacement of bridge [188], (iv) *Emission Event Counts*: 20 loading cycle hits describing crack extension, closure, and surface rubbing [189]. (**c**) Sensitivity of frequency domain features to damage: (i) *Temperature*—Thermal Wave Modulation: infrared phase images of plate defects with a modulated heat source [183], (ii) *Strain*—PZT Conductance: from compression of RC beam [190], (iii) *Acoustic*—Spectrum Analysis: Gaussian mixture model for crack classification [191]. (**d**) Sensitivity of frequency domain features to environment: (i) *Electrochemical*—Impedance Spectra: Nyquist, Bode plots, and equivalent circuits for identifying steel corrosion in concrete [186]; (ii) *Ultrasonic*—FFT Coefficient: Lamb wave extraction of surface crack [187]; (iii) *Visual*—Power Spectral Density (PSD): vision sensor displacement measurements of pure beam [188]; (iv) *Vibration*—Mode Shapes: of health and damaged aluminum truss [192].

**(C4.4) Low Sensitivity to Environmental and Operating Conditions:** IRT errors exceed 20% under fluctuating environmental conditions [179], while electrochemical DTs experience over 40% shifts in galvanic current due to pH and temperature changes [131]. Strain-based FBG sensors require minimal thermal compensation in steel and composites due to low thermal expansion coefficients [3]. UGW suffer near-field distortion above 50 °C, and AE sensors require 1000× amplification to filter environmental noise [7]. Vibration-based SHM undergoes 14–18% modal frequency shifts under seasonal changes [12], while camera-based SHM exhibits moderate brightness fluctuations, affecting accuracy [81,180]. Among these, FBG and AE sensors are the most robust, while IRT and electrochemical methods require stricter environmental controls. Several time and frequency features are evaluated with respect to damage and environmental criteria (C4.1 and C4.4) in Figure 9.

**(C4.5) Sufficient Dynamic Range for the Features to Accommodate a Range of Damage Sizes:** Ultrasonic DTs detect 2.77 mm defects, analyzing guided wave Root Mean Square Deviation Damage Index [152]. AE sensors capture sub-millimeter crack initiation via 100 kHz–1 MHz wave monitoring [7]. Strain-based FBG sensors cover deformation spectra from microstrains to thousands of microstrains, accommodating both localized and large-scale damage [3]. Vibration-based DTs span 0.1 Hz to several kHz, resolving both global deformations and high-frequency anomalies [154,181]. IRT identifies defects up to 6 mm deep, while advanced techniques like pulsed thermography reach 4 mm in composites [51]. Camera-based SHM detects 0.1 mm displacements from 175+ meters [81]. Among these, ultrasonic and AE techniques offer the broadest dynamic range, while FBG, vibration, and IRT methods specialize in different damage scales.

**(C4.6) Providing Reliable Damage Evolution Monitoring:** AE reliably identifies different wearing mechanisms within the frequency range [300 kHz, 900 kHz]. Strain-based FBG sensors provide 1 μ*ε* resolution, detecting early-stage fatigue [60]. Vibration-based SHM captures 0.1 Hz shifts in frequency, tracking progressive degradation [169]. IRT identifies subsurface fatigue cracks with high accuracy, though the heating times and intensity can produce deviations of up to 35% [51]. UGW detects 0.5 mm fatigue cracks in metals, offering high sensitivity [182]. ML integration enhances AE and ultrasonic DTs, improving crack growth classification accuracy to >90% [179]. Among these, AE and UGW provide the best real-time damage tracking, while electrochemical sensors excel in corrosion monitoring.

**Figure 10:**
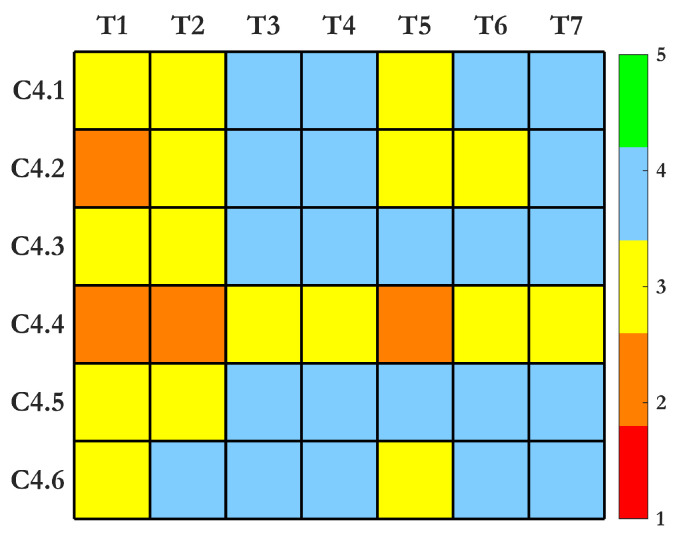
Subcriterion heatmap scores based on features sensitivity.

The heatmap in Figure 10 reveals a balanced view of SHM feature sensitivity across techniques. UGW and AE methods continue to show strong performance in detecting damage, tracking damage evolution, and handling a broad dynamic range. Strain-based (FBG) sensors maintain high scores in consistency, resolution, and monitoring but face moderate environmental sensitivity. Electrochemical methods are effective for corrosion monitoring but are hindered by environmental fluctuations. Thermal (IRT) methods, though capable of detecting surface defects, suffer from low reliability under uncontrolled conditions. Visual and vibration-based techniques perform reasonably well but require precision alignment and calibration to minimize errors. This analysis underscores the need to select SHM methods based on both structural requirements and environmental constraints for optimal sensitivity and reliability. The advantages and drawbacks of each method are summarized in Table 5.

**Table 5:** Features sensitivity—summary of methods: published literature, limitations, and advantages.

Method	Limitations	Advantages
(T1) Temperature [12,51]	Affected by ambient conditions Surface reflections and emissivity variations Requires controlled environment	Highly sensitive to thermal anomalies Effective for detecting delaminations and debonding
(T2) Electrochemical [131,150,193]	Sensitive to temperature and humidity Requires calibration and control Challenged by electromagnetic interference (EMI)	Effective for real-time corrosion monitoring Can be shielded against EMI
(T3) Strain [3,156,163]	Sensitive to temperature variations Requires strategic sensor placement High computational demand	Captures deformation characteristics Long-term reliability of FBG sensors
(T4) Ultrasonic [72,152,182]	Influenced by boundary conditions Requires experienced operators Limited by structural shape	High sensitivity to internal defects Reliable subsurface discontinuity detection
(T5) Visual [81,153]	Affected by lighting conditions Requires stationary camera setup	Sensitive to visual anomalies High precision in displacement measurement
(T6) Vibration [12,154,180]	Influenced by temperature, humidity, and wind loads Requires data filtering	Captures dynamic structural changes High sensitivity with piezo accelerometers
(T7) Acoustic [4,7,8]	Susceptible to external noise and vibrations Complexity in signal interpretation	Early damage detection Real-time distinction of AE events

### 4.5. (C5) Criterion 5: Digital Twin Simulation

**(C5.1) Modeling Accuracy and Predictive Capability:** DT models integrated with Finite Element Methods (FEM) achieve <5% error in stress–strain estimations, with multi-physics models improving predictive accuracy [3]. Temperature-based DTs detect defect depth with an accuracy of 2% [51], though environmental sensitivity affects long-term reliability [12]. Electrochemical DTs successfully track pitting and crevice corrosion in concrete by quantifying open circuit potential with >90% accuracy, incorporating redox reaction kinetics to refine predictions [131,194]. Strain-based DTs using FBG sensors provide 1 μ*ε* resolution, enabling real-time deformation monitoring [156,195]. Ultrasonic DTs detect 2.77 mm defects, achieving > 95% success rates via guided wave scattering models [172]. Visual DTs resolve 0.1 mm displacements, offering scalable anomaly detection [81]. Vibration-based DTs maintain > 0.98 correlation between experimental and numerical mode shapes [154,196]. Among these, strain-based, vibration-based, and ultrasonic sensing offer the highest modeling accuracy at a reasonable computational cost.

**Figure 11:**
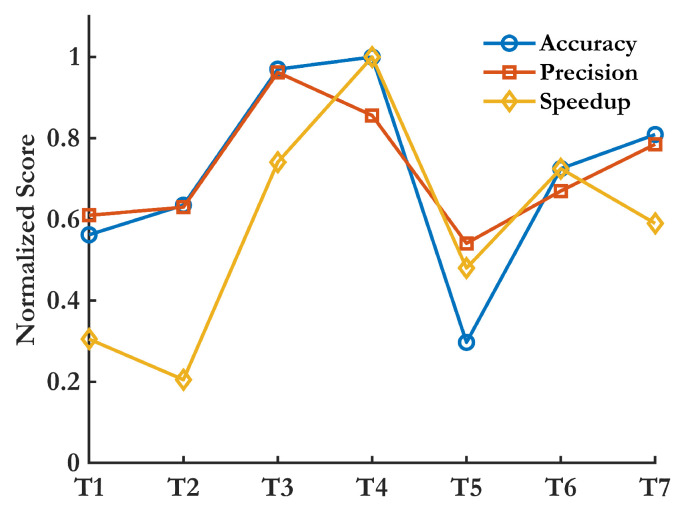
Sensing technologies (T1–7) integrated within a Digital Twin framework—normalized accuracy, precision, and speedup performance.

**(C5.2) Computational Efficiency and Scalability:** SHM-based DTs demand high computational efficiency, particularly for wave propagation models requiring > 10 h on HPC clusters [197]. Extended FEM-Level Set Method reduces computational costs by 30% [198], while Wave Finite Element Methods (WFEM) improve large-scale defect detection efficiency by 50% [199]. Scaled Boundary FEM (SBFEM) enhances strain-based DTs [3] and vibration modeling, reducing computational costs by 40% [200]. Visual DTs using DL accelerate 3D reconstruction by 40%, though data storage remains a limitation [201]. Acoustic sensing-based DTs leveraging edge computing reduce processing load by 65%, improving scalability [202]. Among these, ultrasonic, vibration-based, and strain-based methods maximize computational efficiency, while edge-computing-enhanced AE DTs offer the best scalability [203]. The accuracy, precision (C5.1), and speedup (C5.2) are normalized and illustrated across the seven technologies in Figure 11.

**(C5.3) Integration with Experimental and SHM Data:** DTs improve predictive accuracy by integrating experimental and SHM data, allowing real-time updates [204]. Strain-based DTs reduce FEM uncertainty by 13.5–16.8% through empirical strain gauge data [205]. Ultrasonic DTs enhance defect localization by 95%, though environmental noise necessitates filtering [172]. Visual DTs reconstruct 3D structures at 0.1 mm resolution, enabling automated anomaly detection [206]. Vibration-based DTs maintain > 0.98 correlation with real-time modal data [207]. Among these, strain-based, vibration-based, and ultrasonic methods provide the most accurate DT updates, while visual and AE methods ensure superior real-time SHM integration.

**(C5.4) Versatility Across SHM Techniques:** DTs support multiple SHM techniques, from camera-based 3D reconstructions using LiDAR and photogrammetry to vibroacoustic twins optimized for seismic monitoring [45]. Strain-based DTs provide 1 μ*ε* resolution, making them effective across aerospace and civil structures [148]. Ultrasonic DTs identify 2.77 mm defects, particularly in composite and metallic structures [72]. Semi-Analytical FEM and Spectral Element Methods enable real-time stress localization, improving defect tracking [177]. AE DTs classify cracks with >90% accuracy, though external noise remains a challenge [7,208]. Among these, strain, ultrasonic, and vibration-based sensing provide the highest versatility, while visual and AE methods excel in large-scale and real-time SHM.

**(C5.5) Robustness to Real-World Imperfections:** Environmental uncertainties and sensor noise challenge DT reliability. Thermal wave modulation in temperature-based DTs detects defects with ±6.37% error, though recalibration is required due to ambient fluctuations [52]. Electrochemical DTs often require adaptive filtering to compensate for moisture sensitivity [131,194]. Ultrasonic DTs mitigate noise through Singular Value Decomposition (SVD), improving defect detection reliability by 40% [209]. Vibration-based DTs using Dynamic Condensation and Domain Decomposition improve robustness, reducing environmental influence by 30% [154,210]. DTs utilizing Spectral and Boundary Element Methods (SEMs, BEMs) reduce convergence errors in physical *S*_0_ and *A*_0_ modes, though external vibrations can still degrade signal clarity [211]. Among these, ultrasonic, vibration-based, and strain-based methods provides the highest robustness, while BEM and SVD filtering enhance DT reliability.

**(C5.6) Ease of Implementation and Cost-Effectiveness:** DT scalability varies based on sensor deployment and integration complexity. Temperature-based DTs offer cost-effective anomaly detection but require periodic recalibration [12]. Electrochemical DTs require comparatively higher computational resources, limiting real-time scalability [150]. Ultrasonic DTs balance cost and resolution but require higher data storage than visual-based methods [212]. Visual DTs reconstruct 3D models at 0.1 mm resolution, but hardware costs exceed USD 10,000 per system [81]. Vibration-based DTs reduce long-term monitoring costs by 40% compared with strain-based methods [213]. AE DTs offer cost-effective scalability, though they remain more sensitive to noise than vibration-based approaches [7]. Among these, vibration and AE-based DTs provide the best cost-effectiveness, while visual and ultrasonic DTs maximize accuracy at higher expense.

**Figure 12:**
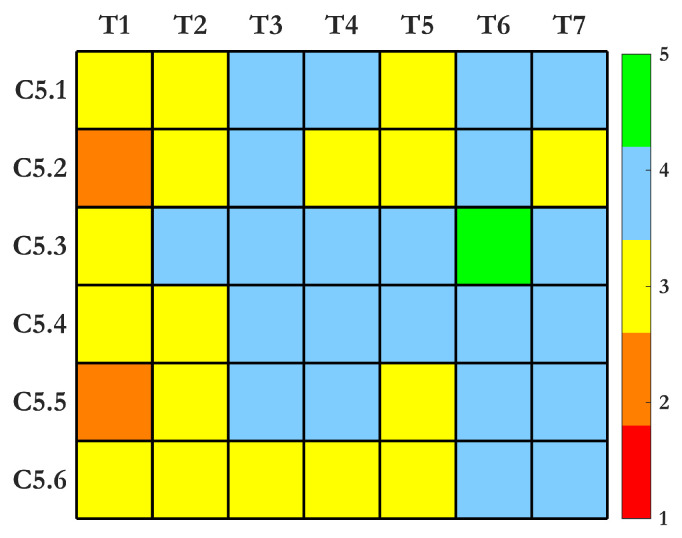
Subcriterion heatmap scores based on DT integration.

The heatmap in Figure 12 highlights the strengths and trade-offs of SHM techniques in DT simulations. Strain, vibration, and ultrasonic methods lead to modeling accuracy, integration with experimental data, and robustness, making them suitable for precision-critical applications. Vibration-based models excel in real-time updates and scalability, while AE techniques balance accuracy and cost-effectiveness through edge computing. Thermal and electrochemical methods, though less robust, offer easy implementation and cost advantages for large-scale deployments. Visual models provide versatile, high-resolution data but face storage and processing limitations. Overall, the optimal SHM approach depends on project-specific needs, with hybrid systems offering the best combination of performance and scalability. The advantages and drawbacks of each method are summarized in Table 6.

## 5. Global Synthesis of Performance Scores for SHM Technologies

**Figure 13:**
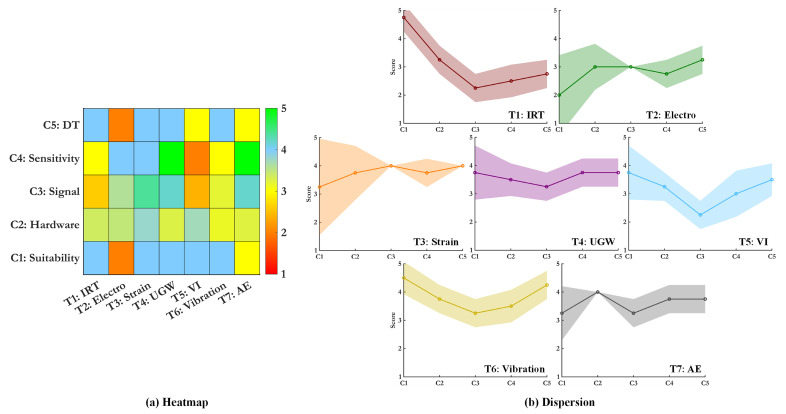
Global synthesis of performance scores for SHM technologies based on C1–C5. (**a**) heatmap displays the mean scores computed from subcriteria, (**b**) dispersion plots show the mean performance (solid lines) and variability (envelopes representing standard deviation) for each technology across criteria.

The detailed quantitative discussions in Section 4 are now synthesized into a global performance heatmap and corresponding dispersion plots (Figure 13). The heatmap illustrates how each technology performs across C1–5, with scores calculated by taking equal-weighted means of the subcriteria. The accompanying dispersion plots provide additional insights, with solid lines representing the mean scores and envelopes denoting the standard deviation, which encodes the variability in performance across the subcriteria.

The heatmap and plots confirm that no single method excels universally, emphasizing the importance of context-specific deployment strategies. Key observations from the dispersion plots reveal that strain sensing, UGW, and vibration-based methods demonstrate relatively consistent performance across criteria, indicated by flatter mean curves and narrow standard deviation envelopes. These methods balance deployability with diagnostic capabilities effectively. Particularly, strain sensing maintains a high mean sensitivity score of 4, enabling it to detect frequency shifts with an error margin of ±0.5%, suitable for long-term monitoring of large structures. UGW scores 4 on signal characteristics and maintains moderate hardware complexity (score of 3), making it highly reliable for both surface and subsurface diagnostics, with the ability to detect delaminations as small as 3.5 mm with 95% confidence. In contrast, methods such as VI and IRT score consistently well in deployment-related criteria (suitability and hardware, ≈4), owing to their ease of installation. However, these techniques exhibit significant performance variability in sensitivity and diagnostic reliability. The dispersion envelopes reveal higher variability in subsurface damage detection (mean scores of 2.5), reflecting their susceptibility to environmental factors.

Specialized methods like electrochemical sensing and AE demonstrate strong performance in specific contexts but exhibit considerable variability in hardware and environmental integration. Electrochemical sensing, with a mean sensitivity score of 4, excels at localized corrosion monitoring and reduces maintenance costs by 9.1%. However, the large dispersion in hardware scores highlights the challenges posed by frequent recalibration due to sensor drift (3.2% monthly). Similarly, AE achieves strong sensitivity (mean of 4) for early-stage crack detection with an SNR exceeding 20 dB, though its performance is hindered by noise interference, reflected by wide dispersion in signal quality scores.

The dispersion plots also highlight notable pinch points—criteria where certain technologies exhibit sharp performance drops or variability spikes—indicating areas for targeted improvement. These observations emphasize the potential benefits of hybrid strategies, which combine large-scale deployable techniques, such as ultrasonic and visual inspection, with highly sensitive localized methods like AE or electrochemical sensing. Such hybrid approaches can better balance trade-offs between hardware complexity, sensitivity, and scalability, leading to more comprehensive and adaptable SHM systems.

### Future Outlook and the Need for New-Generation Technologies

While existing sensing technologies have enabled significant progress in SHM, several limitations, as summarized in Table 7, prevent their full potential from being realized. A key issue is environmental sensitivity. Techniques such as IRT and electrochemical sensing are prone to interference from ambient conditions, including temperature fluctuations, humidity, and surface reflectivity. These variations can obscure damage signals and increase the likelihood of false-positive detections, thereby reducing system reliability. Similarly, vibration-based monitoring techniques face signal degradation caused by environmental noise and operational disturbances, requiring extensive signal processing and filtering to maintain accuracy. Coverage and scalability present additional challenges. Strain-based sensing methods, for example, require dense sensor networks to achieve adequate spatial resolution over large structures. This increases the complexity and cost of both installation and maintenance. Likewise, ultrasonic and acoustic techniques, though capable of detecting internal damage with high sensitivity, demand precise placement and configuration of sensors, along with significant technical expertise for interpretation. These requirements complicate their deployment, particularly in large, complex, or remote infrastructure projects.

Signal quality and data management are persistent concerns for many SHM methods. Technologies such as AE and UGW generate extensive data streams that are difficult to process in real time. Visual inspection techniques that rely on automated image processing also face similar challenges, as they require a large storage capacity and computational resources to handle high-resolution data efficiently. Without optimized data integration and storage strategies, the scalability of these methods remains limited. Moreover, integrating these sensing technologies with DT frameworks has proven difficult due to high computational demands. Real-time data assimilation and model updating, which are essential for predictive maintenance, are resource-intensive and particularly challenging for techniques that produce complex, high-frequency data. This limits the applicability of DTs in scenarios where immediate feedback is required.

Given these constraints, future SHM advancements must address these limitations by enhancing robustness, scalability, and data efficiency. Section 6 will delve into how emerging technologies, including automation, multisensory integration, and real-time simulation, are uniquely positioned to overcome these challenges. These next-generation advancements promise to mitigate the environmental sensitivity, signal processing demands, and scalability issues faced by classical methods. Innovations such as self-sensing structures and IoT-enabled data fusion can revolutionize SHM by providing adaptive, remote, and real-time monitoring solutions. By reducing the dependency on dense sensor networks, minimizing false positives, and improving dynamic data assimilation, these technologies aim to transform structural maintenance from reactive to predictive, enabling more efficient and resilient infrastructure management on a global scale.

## 6. New Generation Technologies Enhancing SHM Systems

New-generation sensing technologies (NGs) encompass the latest advancements in sensor technologies, aiming to overcome the limitations of conventional sensors by providing more accurate, reliable, and efficient data for assessing and monitoring civil structures. In this regard, five emerging sensing technologies are presented in the section:

- **NG1:** UAV/robot-assisted sensing;
- **NG2:** Smart self-sensing structures;
- **NG3:** Advanced multisensory data fusion-aided sensing technologies;
- **NG4:** Advanced Digital Twin-driven sensing;
- **NG5:** Wireless/IoT-based sensing.

**Figure 14:**
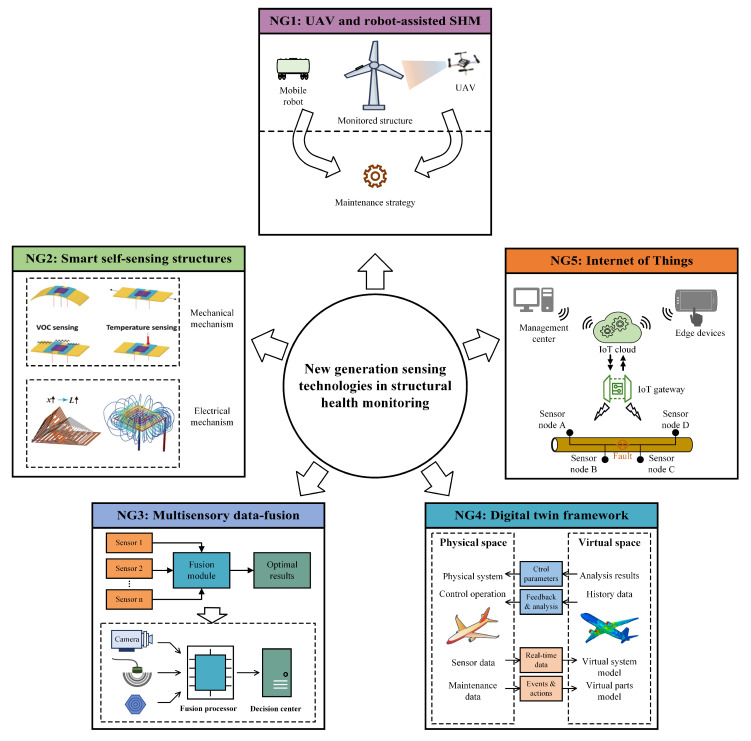
New generation technologies in structural health monitoring [215].

These new-generation SHM technologies are graphically illustrated in Figure 14 and are subsequently discussed in detail in the following subsections.

### 6.1. (NG1) UAV-Assisted and Robot-Based Monitoring Systems

Over the past few years, there has been a surge of interest in the realm of predictive maintenance surrounding drones, alternatively referred to as UAVs [216,217,218,219,220,221,222,223,224,225,226,227,228,229,230,231]. For monitoring purposes, particularly T5: Visual Inspection, as described in Section 4.5, UAVs are equipped with an array of sensors and cameras, enabling data collection and a wide range of tasks. They excel at detecting displacement, measuring deflection, and gathering relevant data through photogrammetric techniques and CV algorithms. By incorporating sensors such as RGB cameras or Light Detection and Ranging (LiDAR), UAVs can capture high-resolution image data with exceptional spatial precision. Another notable advantage lies in their capacity to access and examine locations that may pose difficulties or hazards for human access. These technologies can independently navigate vast structures, such as bridges, capturing data from multiple angles and viewpoints—thereby enabling a comprehensive evaluation of structural integrity and the identification of potential defects or damage.

Nevertheless, there are certain challenges preventing the efficient deployment of UAVs within SHM frameworks. One such challenge pertains to the accuracy and sensitivity of the sensors onboard these UAVs. The quality of data gathered is contingent on the stability and movement of the UAV itself, with adverse weather conditions and vibrations potentially affecting data accuracy. Ensuring precise measurements necessitates careful consideration. Additionally, there are policy-related obstacles that impede research in robotics-based remote sensing for SHM. Regulatory frameworks, including the Federal Aviation Administration’s Code of Federal Regulations Part 107 [232], impose limitations on UAV operations, encompassing line-of-sight requirements and constraints on the concurrent operation of multiple UAVs. Furthermore, challenges related to hardware and software integration range from extending the battery life for longer flight durations to designing the capability of withstanding adverse weather conditions and ensuring robust wireless communication in remote areas. Technological advancements in UAVs, including multi-rotor UAVs with stable positioning and improved visual navigation systems, have already expanded their capabilities in SHM.

The use of robots in remote sensing and monitoring opens up the opportunity to inspect large-scale structures like bridges, dams, and oil and gas installations, even if they span vast distances or are situated in hazardous environments. This application empowers engineers and researchers to gather critical real-time data regarding the health of these infrastructure components without the need for labor-intensive, expensive, and potentially perilous manual inspections. These robots come equipped with an array of sensors, including visual cameras, LiDAR scanners, and ultrasonic sensors, enabling them to identify and measure a wide range of parameters, including cracks, deformations, vibrations, and temperature fluctuations. The sensors capture data from the structure and transmit it to a central data collection system for subsequent processing and analysis.

One particularly notable advantage of robotics-based remote sensing and monitoring is its ability to provide real-time data [233]. This continuous monitoring enables engineers to promptly identify and respond to changes in the structural health of a system, resulting in more effective maintenance and mitigation strategies. Real-time data also facilitates the detection of sudden or unforeseen events, such as structural failures or extreme environmental conditions. Furthermore, the use of robotics in remote sensing and monitoring minimizes the necessity for human intervention, as these robots can autonomously navigate and inspect structures [233,234]. This not only decreases the potential for human error during data collection but also mitigates safety risks for inspection personnel, especially in high-risk environments [235]. In this way, these technologies can greatly reduce the operation and maintenance costs of large structures, such as offshore wind turbines, by automating the inspection process. Despite these significant advantages, challenges remain in implementing robotics-based remote sensing and monitoring in practical applications. These obstacles encompass the development of dependable and robust robotic platforms capable of maneuvering through complex environments, the integration of diverse sensing technologies into a unified system, and the advancement of sophisticated algorithms for data interpretation and decision-making. More specifically, recent advances in AI, particularly deep learning [236], have enabled robots to process high-dimensional sensor data for real-time anomaly detection, using convolutional neural networks (CNNs) to identify localized structural damage from visual or vibrational inputs [143]. Reinforcement learning (RL) further enhances robotic autonomy by optimizing navigation and inspection paths in dynamic environments, enabling adaptive decision-making under uncertainty. Deep reinforcement learning (DRL) frameworks [237] are also being explored to unify perception, control, and predictive maintenance, allowing robots to learn long-term inspection strategies while balancing efficiency and accuracy.

These developments prove highly pertinent to addressing the limitations (see Table 7) posed by technologies T1–7 in relation to the five criteria C1–5 proposed in this work. They are summarized below in Table 8 with relevant references to the state-of-the-art literature.

### 6.2. (NG2) Smart Self-Sensing Structures

Engineering structures can be made more efficient and functional with innovative smart structural materials, which represent a groundbreaking category of structural components imbued with remarkable functionalities that allow for autonomous internal health inspection. Their growing popularity stems from an intrinsic design featuring self-sensing, self-healing, and self-actuation capabilities, which provide critical insights into the internal dynamics of the structures [241,242,243,251,268,269,270,271]. Flexible smart sensing skin technology can continuously evaluate the condition of infrastructures such as buildings, bridges, or aerospace structures [241,242,243,251,268,269]. These adaptable and real-time sensors excel at detecting cracks, monitoring strain, vibration, and temperature fluctuations, and identifying defects such as corrosion and material aging. This makes them highly valuable in addressing the shortcomings of T1: IRT, T3: strain, and T5: vibration-based approaches. Further, they provide valuable data for fatigue analysis, assess the impact of environmental conditions, and ensure safety during seismic events or adverse weather. The data collected enable predictive maintenance models, allows for remote monitoring, and reduces the need for costly manual inspections.

**Figure 15:**
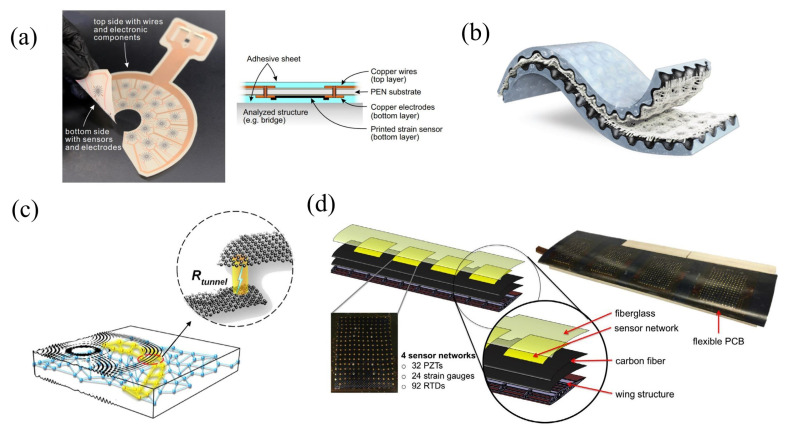
Novel sensor technologies for the development of self-sensing structures. (**a**) Integrated strain sensor array on a flexible printed circuit board for SHM. Reprinted from Ref. [272]; (**b**) An illustration of the stretchable nf-SPENG (Nanofiber-based Stretchable PiezoElectric NanoGenerators) equipped with graphite electrodes and nanofiber mats. Reprinted from Ref. [269] with permission; (**c**) Schematic of guided ultrasonic wave self-sensing in the nanoparticle-formed percolating network operating based on change in local electrical resistance. Reprinted from Ref. [242] with permission; (**d**) Intelligent composite wing with self-sensing capabilities. There are 148 micro-sensors embedded in this composite layup, including 32 piezoelectrics, 24 strain gauges, and 92 RTD sensors. The composite wing consists of layers, flexible PCBs, and four networks. Reprinted from Ref. [241] with permission.

Practical implementations of smart sensing skin technology are depicted in Figure 15. Figure 15a illustrates the schematic concept of a proposed SHM system integrating ultra-thin piezoelectric strain sensors on a polyethylene naphthalate substrate [272]. In Figure 15b, S. Siddiqui et al. [269] present the SPENG, which uses piezoelectric nanofibers and a flexible graphite electrode on a 3D micropatterned substrate. This technology efficiently harvests energy from human motion, making it suitable for wearable electronic systems. Y. Li et al. [242] explore the use of nanocomposite sensors and “self-sensing” composites for SHM, as illustrated in Figure 15c. The approach integrates graphene nanoparticles within fiber-reinforced polymer matrices to autonomously detect UGW, minimizing intrusion while maintaining precise monitoring of structural integrity. Figure 15d illustrates a smart composite wing developed by F. Kopsaftopoulos et al. [241], equipped with 148 micro-sensors, including piezoelectric, strain gauges, and Resistance Temperature Detector (RTD) sensors. This composite wing demonstrates advanced self-sensing capabilities, enhancing structural monitoring efficiency.

Flexible sensors based on alternate sensing mechanisms also demonstrate great potential for SHM applications [215]. These sensors operate by detecting variations in resistance, capacitance, or inductance caused by surface deformations or internal changes within the monitored structures. Ultimately, smart structural materials harbor the transformative potential to transform structures into sophisticated sensor networks analogous to biological systems. The key contributions of self-sensing structures, compared with established sensing techniques, are summarized in Table 8. While research within this domain is both vibrant and ongoing, substantial efforts are necessary to overcome the challenges pertaining to deployment, scalability, costs, and signal processing, paving the way for a seamless transition of this technology and its widespread adoption in the field of SHM.

### 6.3. (NG3) Advanced Multisensory Data Fusion Techniques

The primary objective of multisensory data fusion (MSDF) is to integrate different sensor types, whether homogeneous or heterogeneous, to enhance accuracy in damage detection and identification in the realm of SHM [273]. A major challenge lies in the complexity of processing and merging data from multiple sensors. Based on the level of processing performed on the data, there are typically three categories of fusion: data-level fusion, feature-level fusion, and decision-level fusion [274]. Data-level fusion is the process of combining raw sensor data from various sources to create a unified and comprehensive dataset. Feature-level fusion, the following stage, involves integrating features extracted from the raw data to produce a composite feature. Lastly, decision-level fusion deals with higher-level aggregation from multiple classifiers or decision-making systems.

Common approaches used within MSDF include DL-based fusion [245,275], Kalman/ particle filtering [244,276,277], image fusion [278,279], Bayesian fusion [274,280], and Dempster–Shafer (DS) theory [281]. DL-based fusion can be applied at all three presented levels, where raw data, extracted features, or output decisions are concatenated using deep neural networks. For instance, the transformer networks employ attention mechanisms to dynamically align and integrate heterogeneous sensor data by capturing cross-modal dependencies across spatial and temporal domains, enhancing robustness to noisy or missing data in complex environments [282]. Kalman/particle filtering is typically employed in real-time data-level fusion for continuous measurement sources, such as in accelerometer and GPS fusion [283]. However, Kalman filtering is restricted to combining measurements disturbed with Gaussian noise, while particle filtering accommodates measurements with non-Gaussian noise. Image fusion has become a recent research focus due to the advancements in artificial intelligence and image-based inspection (i.e., NG1: UAV-based inspection in SHM). It can be categorized into pixel-level fusion, feature-level fusion, and decision-level fusion [284], where pixel-level corresponds to data-level fusion by performing data-level fusion on image pixels. Lastly, Bayesian fusion and DS theory fall under the category of probabilistic fusion. While Bayesian fusion can be applied across all three levels of fusion, DS theory is typically employed at the decision level.

Recent studies in MSDF, summarized in Table 8, highlight significant advancements in performance criteria. Beyond these improvements, MSDF is poised for integration into NG4: DT 2.0 and NG5: IoT environments, which will be discussed in detail in the following subsection. In IoT frameworks, MSDF addresses challenges associated with distributed and heterogeneous systems, including nonlinear components, time-varying sensing processes, and the need for standardized communication protocols. Over the past two decades, MSDF has enabled predictive maintenance in remote areas by utilizing parallel algorithms and localized data fusion supported by sensors and microprocessors.

In DT frameworks, MSDF plays a crucial role in real-time interaction between physical entities and their virtual models. Synchronized data from onboard sensors and offline NDT establish baseline references. Edge or fog computing enhances this process by handling raw sensor data locally, improving signal quality through data-level fusion to enhance SNR and fidelity while archiving historical records for future analysis. This integration supports adaptive physics-based and data-driven models within the DT, dynamically updating with fused data to reflect changing environmental conditions. By merging real-time monitoring with NDT baselines, MSDF provides a robust data foundation for AI algorithms. This enables the identification of trends, correlations, and predictive insights, ultimately supporting more informed and proactive decision-making for critical infrastructure management.

### 6.4. (NG4) Digital Twin Framework—DT 2.0 and (NG5) Internet of Things

**Figure 16:**
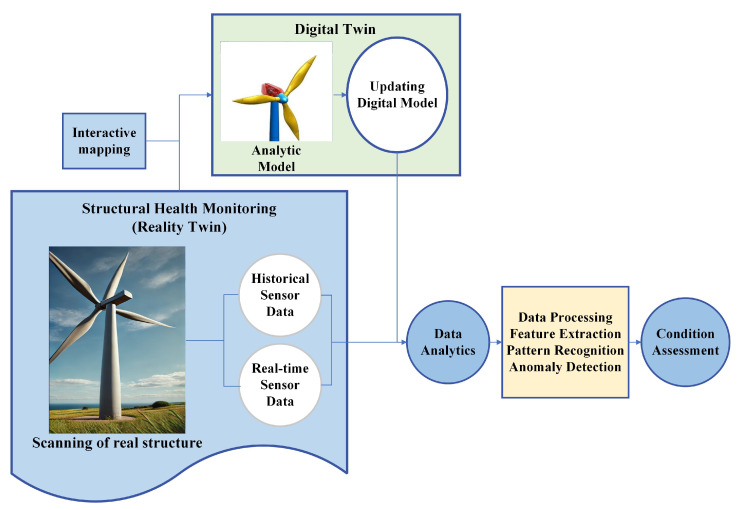
The functional schematic of the concept of DT in SHM. A virtual replica of a physical structure or system is created and continuously updated with data from real-time sensors. In this way, the behavior and performance of the physical structure can be analyzed and simulated in a virtual environment.

Industry 4.0 is marked by the shift towards the digitization of infrastructure and manufacturing systems, driven by continuous data exchange and predictive technologies such as 5G and IoT [209]. Central to this transformation are DTs, which create virtual replicas of physical systems that synchronize in real time with sensor data. These replicas enable performance monitoring, scenario exploration, and early issue detection through AI-driven insights [121], as shown in Figure 16. DT technology has demonstrated significant benefits across various industries. NASA’s early use of DTs during the Apollo 13 mission has evolved into applications like GE Power’s ‘digital wind farms’, achieving over 20% efficiency gains [285,286]. In civil infrastructure, CS Shim et al. [287] developed a DT model for bridge maintenance, integrating 3D models with real-time inspection systems, while M. Omer et al. [257] leveraged LiDAR and virtual reality for enhanced bridge inspections. In aerospace, Eric J. Tuegel et al. [288] reengineered structural life prediction models using DTs, while BR Seshadri and T. Krishnamurthy applied guided wave responses and genetic algorithms for in-flight structural monitoring [289].

It is important to distinguish between two concepts related to digital twins in this article. *C5: Digital Twin Integration* is an evaluation criterion that assesses the capacity of current sensing technologies to provide real-time, synchronized data for updating digital models. This criterion focuses on a technology’s ability to facilitate integration with existing DT systems for real-time tracking and diagnostics. In contrast, *NG4: New-Generation Digital Twin (or DT 2.0)* refers to the ongoing evolution of DT technology that incorporates advanced IoT, ML, and real-time simulation capabilities. NG4 aims to overcome current SHM limitations, such as low sensitivity, poor SNRs, and scalability challenges, by leveraging continuous data fusion and predictive analytics.

**Figure 17:**
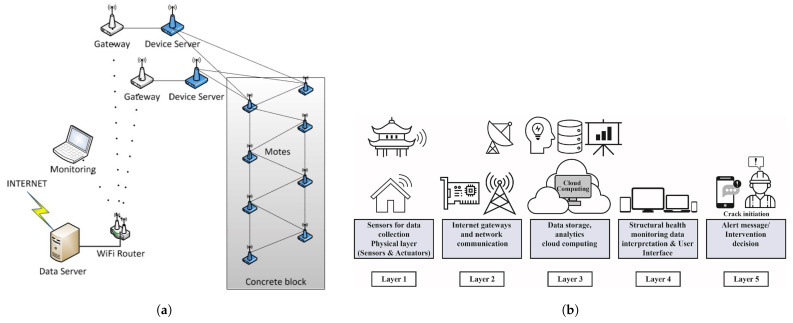
Wireless smart SHM systems. (**a**) Wireless sensor network monitoring system scheme based on the work conducted by J. Cabezas et al. Reprinted from Ref. [290]; (**b**) the five-layer architecture of IoT-based monitoring systems. Reprinted from Ref. [249] with permission.

The integration of IoT and wireless smart sensors has significantly enhanced the remote monitoring of physical, chemical, mechanical, and environmental properties in structures, as extensively documented [249,264,290,291,292,293,294,295,296,297,298,299,300]. These sensors, widely used in civil and aerospace SHM, offer reduced costs, easy deployment in remote areas, and enhanced data transmission through compression techniques [11,290]. They support multimetric sensing for conditions such as strain, temperature, and vibration while conserving battery life with event-triggered sensing and efficient edge-computing algorithms [249,301]. IoT-based SHM systems employ a multi-layer architecture where sensors collect data, gateways transmit it, and cloud platforms analyze it to provide feedback for system optimization [249]. Integration with cyber-physical systems enhances real-time data collection and reduces maintenance costs for structures like buildings and bridges [249,250]. Additionally, Franchi et al. emphasize the role of 5G technology in disaster management, particularly for low-latency communication in earthquake early warning systems [250]. As shown in Figure 17, the wireless sensor network monitoring system and the five-layer architecture of IoT-based monitoring systems demonstrate the integration of various technologies for structural health monitoring. As shown in Figure 17, the wireless sensor network monitoring system and the five-layer architecture of IoT-based monitoring systems demonstrate the integration of various technologies for structural health monitoring.

The integration of DT and IoT technologies has also significantly advanced SHM by addressing limitations such as low sensitivity, poor signal-to-noise ratios (SNRs), and hardware complexity. DTs act as virtual replicas, continuously synchronized with real-time data from IoT sensors, enhancing sensitivity and enabling the early detection of anomalies previously missed by traditional methods [302]. Wireless sensor networks simplify deployment and reduce hardware costs, while their robust data streams allow DTs to dynamically refine predictive models, minimizing physical maintenance and intrusion. This adaptability extends to diverse structures—bridges, buildings, aircraft, and wind turbines—where DTs simulate future structural conditions for predictive maintenance, reducing disruption and costs. Future IoT-DT integration is expected to include advanced machine learning (ML) analytics capable of processing large datasets to detect early structural wear, making SHM more efficient, proactive, and scalable, thereby ensuring structural longevity and high returns on investment [248]. The contributions of DT and IoT technologies to recent advancements in SHM are summarized in Table 8, using the five performance criteria C1–5.

Recent advancements in ML and AI, integrated with digital twin frameworks and IoT sensor networks, have significantly enhanced structural health monitoring by enabling automated data analysis, anomaly detection, and predictive maintenance. Real-time data from IoT sensors continuously updates digital twin models, providing accurate simulations of physical systems. Deep learning architectures, including CNNs and LSTMs, have proven effective for damage detection in complex environments [303,304]. Hybrid frameworks that merge physics-based models with digital twins and data-driven insights further optimize sensor placement and degradation prediction [305,306]. Additionally, reinforcement learning is being explored for adaptive maintenance strategies to reduce downtime [307]. Enhanced computational capabilities and open access datasets are accelerating AI integration into SHM systems, leading to more resilient infrastructure management [308,309].

## 7. Concluding Remarks

### 7.1. Summary

This review presents a comprehensive exploration of state-of-the-art sensing technologies for both diagnostic and prognostic applications in structural health monitoring (SHM). In Section 4 and Section 5, we systematically evaluated seven major sensing technologies—visual inspection, vibration response analysis, acoustic methods, strain measurement, ultrasonic testing, temperature sensing, and electrical/electrochemical techniques—against five performance criteria: deployment suitability, hardware requirements, signal characteristics, sensitivity, and DT integration. Unlike previous studies that often rely on qualitative assessments or isolated technology reviews, we adopted a novel approach by assigning objective performance scores. This method emphasizes transparency and avoids rigid rankings, recognizing that each technology is highly tailored to specific operational contexts.

In Section 6, we further examined the integration of emerging technologies, including MSDF, self-sensing materials, UAVs, IoT frameworks, and next-generation DTs. Our review detailed the complexities of MSDF at data, feature, and decision levels, highlighting the techniques and algorithms that enable these advancements. The integration of MSDF with IoT and DT systems holds transformative potential, enabling remote monitoring, enhanced operational efficiency, and real-time insights into structural performance. These innovations address key limitations of traditional SHM methods, such as low sensitivity and high hardware complexity, by leveraging adaptive, scalable, and predictive capabilities. The findings underscore the pivotal role of both advanced sensing technologies and new-generation systems in reshaping SHM practices. However, critical challenges were identified that must be addressed, including computational complexity, sensor calibration, and feature selection. Overcoming these obstacles will require further research and refinement to fully realize the potential of these technologies in achieving safer, more durable, and cost-effective infrastructure management.

### 7.2. Future Perspectives

Future research should prioritize developing scalable algorithms, improving data fusion techniques, and optimizing sensor integration to enhance real-time decision-making. Concrete future prospects include the following:

- Conducting experimental studies and field validations to validate the effectiveness and practicality of sensing technologies in SHM and assess their performance in real-world applications.
- Further investigate data fusion techniques for multisensory data fusion in SHM systems, focusing on multiple image data-level fusion. Exploring different fusion algorithms, such as spatial domain fusion, frequency domain fusion, and DL-based fusion, to assess their effectiveness in improving the quality and reliability of fused images.
- Improve the hardware requirements for SHM systems, particularly in the context of multiple image data-level fusion. Develop portable and affordable imaging solutions, such as digital cameras and infrared cameras that can be easily deployed for in-situ and automated monitoring of large-scale structures.
- Exploring the integration of wireless smart systems and IoT in SHM. Develop wireless smart sensors for continuous monitoring of structural health parameters and investigate data compression techniques for efficient data transmission and storage. Study the challenges associated with continuous monitoring and resource constraints in wireless smart systems and develop event-triggered sensing techniques to optimize battery life and extend operational lifespan.
- Investigating the application of DT technology in SHM and exploring the integration of DTs with sensor data fusion. Develop advanced algorithms for data fusion in DT environments and study the impact of sensor data on model updating and predictive insights.
- Exploring the potential of ML and artificial intelligence techniques for analyzing and interpreting fused sensor data in DT applications.
- Addressing the computational complexity associated with different sensing technologies and multisensory data fusion to develop efficient algorithms and techniques for data processing and analysis.
- Developing standardized sensor calibration methods for different sensing technologies in SHM to ensure accurate and reliable measurements.
- Refining feature selection techniques to effectively extract relevant information from multisensory data for better anomaly detection and defect identification in structures.
- Practical evaluation of the performance and limitations of new-generation systems in SHM, including their impact on safety, durability, and cost-effectiveness of infrastructure management.

By continuing to refine and adopt these innovations, SHM can advance towards a more predictive and proactive approach, ensuring greater structural longevity, resilience, and sustainability.

## Figures and Tables

**Table 1:** A list of abbreviations used in this article.

**AE**	Acoustic Emission	**MSDF**	Multi-Sensory Data Fusion
**BEM**	Boundary Element Method	**NDT**	Non-Destructive Testing
**CNN**	Convolutional Neural Networks	**PCB**	Printed Circuit Board
**CV**	Computer Vision	**PSD**	Power Spectral Density
**DIC**	Digital Image Correlation	**PWAS**	Piezoelectric Wafer Active Sensors
**DL**	Deep Learning	**PZT**	Lead Zirconium Titanate
**DS**	Dempster–Shafer	**RTD**	Resistance Temperature Detector
**DT**	Digital Twin	**SBFEM**	Scaled Boundary FEM
**FBG**	Fiber Bragg Grating	**SEC**	Soft Elastomeric Capacitor
**FEM**	Finite Element Method	**SHM**	Structural Health Monitoring
**FFT**	Fast Fourier Transform	**SNR**	Signal-to-Noise Ratio
**HPC**	High-Performance Computing	**SPENG**	Stretchable PiezoElectric Nanogenerator
**IoT**	Internet of Things	**SVD**	Singular Value Decomposition
**IRT**	InfraRed Thermography	**UAV**	Unmanned Aerial Vehicle
**MEMS**	Micro Electro Mechanical Systems	**UGW**	Ultrasonic Guided Waves
**ML**	Machine Learning	**WFEM**	Wave Finite Element Methods

**Table 2:** SHM suitability—summary of methods: published literature, limitations, and advantages.

Method	Limitations	Advantages
(T1) Temperature [134,135]	Requires human supervision Limited field of view Environmental impact	Rapid operational efficiency Broad material applicability Reliability, affordability, and user-friendliness
(T2) Electrochemical [56,131]	Just for conductive materials Environmental degradation Data integration challenges	Long-term monitoring Measuring physical, biological, or chemical parameters
(T3) Strain [8,140]	Susceptible to failure and fatigue Local measurement Issues with nonlinearity, hysteresis, and temperature fluctuations	Continuous monitoring Broad structures and materials applicability Providing environmental data
(T4) Ultrasonic [139,141,142]	Complexity in application Impact of structural configuration	Broad material applicability Efficiency and cost-effectiveness Advanced technology integration
(T5) Visual [81,107,143]	Susceptibility to environmental interference Limited by visual conditions sophisticated image processing	Global sensing coverage and automation Advanced technology integration Advanced deployment options (UAVs and robots)
(T6) Vibration [144,145,146]	Sensitivity to environmental noise Complexity and time demands	Continuous monitoring applicability Versatile frequency range Cost-effective and low power
(T7) Acoustic [10,147,148,149]	Scalability and cost concerns Prone to ambient noise Passive operation	Energy efficiency Broad material applicability

**Table 3:** Hardware requirements—summary of methods: published literature, advantages, and limitations.

Method	Limitations	Advantages
(T1) Temperature [47,50,160]	Not reliable for damage located within large depth Largely affected by external environmental condition	Utilization of IR camera is very effective for SHM of moving, large, and inaccessible structural parts
(T2) Electrochemical [56,131]	Susceptible to being affected by sample temperature, spacing, and orientation of the electrodes	Cost-effective setup to assess concrete crack, chloride penetration, reinforcement corrosion, etc.
(T3) Strain [8,155,163]	High cost and inconvenience to cover a large structure	Availability of different sensors with varying accuracy, flexibility, and ease of installation with minimal energy consumption
(T4) Ultrasonic [161,164,165]	Strong expertise regarding placement and attachment of the transducers	PWAS system can be used to develop a fast, simple, lightweight, stable, accessible, safe, low-cost, and smart SHM system to cover a large area
(T5) Visual [45,107,160]	Not reliable for damage located within a large depth	Utilization of image advantageous to identify surface defects in structures like bridges, buildings, turbine blades
(T6) Vibration [116,118,166]	Strong technical expertise regarding placement and attachment of the accelerometers	Cost-effective MEMS accelerometers can be deployed in sensor networks to cover large areas
(T7) Acoustic [7,158,162]	Strong technical expertise, costly data acquisition system, sensors, and analyzing software	AE transducers can be embedded within the structures to monitor health

**Table 4:** Signal characteristics—summary of methods: published literature, advantages, and limitations.

Method	Limitations	Advantages
(T1) Temperature [12,49,50,51]	Expensive for large structures Largely affected by external environmental condition	Qualitative assessment of surface or sub-surface damages
(T2) Electrochemical [131,150,174]	High-quality sensors are costly Infested by external environment Complicated data processing	Effectively assess concrete crack, chloride penetration, reinforcement corrosion, etc.
(T3) Strain [3,151,175]	Strong technical background to compute optimum sensor locations	Non-baseline approaches to assess damages in real-time creation of high-fidelity models using strain maps of structures
(T4) Ultrasonic [164,165,172]	Strong technical background to understand the signal properties Difficulties in discerning the signal properties in the near-field region of the transducers	Capability to identify structural integrity, material integrity, and existing flaws effectively
(T5) Visual [8,87,107]	Computationally expensive for very large structures Strong technical knowledge of image processing and DL	Provide overall damage maps of visible surface damages for large and hard-to-reach structural components
(T6) Vibration [146,166,168,169]	Strong technical expertise regarding signal processing techniques Sometimes infested by environmental and operational conditions	Less complicated in comparison with ultrasonic signals
(T7) Acoustic [10,157,176,177]	Strong technical expertise to interpret and analyze the signal Prerequisite of having baseline data to asses present condition	Capability of detecting a range of damage types, including cracks, delamination, corrosion, and fatigue

**Table 6:** Integration of Digital Twin/FE methods in SHM: limitations and advantages.

Method	Limitations	Advantages
(T1) Temperature [12,51]	High computational cost in DT Complex thermal modeling in FE	Enhanced anomaly detection with FE Advanced thermal simulations in DT
(T2) Electrochemical [131,150,193]	Complex electrochemical modeling Requires high-fidelity DT models	Early corrosion detection in DT Reliable long-term monitoring FE insights into corrosion processes
(T3) Strain [3,156,163]	High resource demand for real-time DT Localized strain challenges in FE Requires precise calibration	High accuracy in deformation prediction Detailed strain distribution in FE Continuous monitoring in DT
(T4) Ultrasonic [72,152,182]	High resolution needed for DT Computationally intensive in FE Complex wave modeling in DT	Enhanced defect detection Real-time updates in DT Effective for large structures
(T5) Visual [45,81,153]	High cost for DT integration Challenges in 3D FE model generation	Advanced structural assessment Detailed FE modeling from visual data Precise anomaly detection in DT
(T6) Vibration [12,154,180]	High computational power needed Challenges in dynamic FE modeling	Real-time structural updates Detailed vibrational analysis in FE Cost-effective monitoring in DT
(T7) Acoustic [4,7,8,214]	Computationally intensive in DT Sensitive to noise in FE analysis Real-time signal processing challenges	Early damage detection Accurate wave analysis in FE Continuous monitoring in DT

**Table 7:** Limitations of SHM techniques across performance criteria.

Technique	C1: Suitability	C2: Hardware	C3: Signal	C4: Sensitivity	C5: Digital Twin Simulation
**T1: IRT**	Requires human supervision and is limited by environmental impact.	High cost for thermal cameras, requires specialized equipment.	Highly sensitive to environmental conditions like temperature and humidity.	Surface reflections and emissivity variations affect sensitivity.	High computational cost; complex thermal modeling in DT.
**T2: Electro**	Limited to conductive materials and affected by environmental degradation.	Frequent recalibration, requires specialized sensors.	Data requires extensive preprocessing to filter environmental effects.	Sensitive to humidity and temperature; EMI challenges.	Requires complex electrochemical models for corrosion tracking.
**T3: Strain**	Susceptible to failure, fatigue, and local measurement challenges.	High installation cost due to sensor network for large structures.	Signal clarity depends on sensor precision and placement.	Temperature-sensitive; high sensitivity to deformation.	Localized strain challenges in DT integration; high calibration needs.
**T4: UGW**	Complexity in application and sensitivity to structural configuration.	Requires strong expertise in placement of transducers.	Signal distortion in near-field regions; requires expert interpretation.	Requires experienced operators to discern defect signals.	Computationally intensive; complex wave simulations required.
**T5: VI**	Susceptibility to environmental interference and limited by visual conditions.	Requires high-resolution cameras and extensive storage.	Poor SNR in noisy environments.	Affected by lighting and positioning conditions.	High cost for integration into 3D FE models.
**T6: Vibration**	Strong expertise needed for signal processing; prone to operational noise.	Requires synchronized accelerometer networks; high storage demand.	Degraded by dynamic environmental changes; requires filtering.	Sensitive to temperature and humidity fluctuations.	Challenges in dynamic DT modeling; high computational demand.
**T7: AE**	Requires baseline data for condition assessment; sensitive to ambient noise.	Costly data acquisition systems; complex signal processing.	Affected by external noise; requires extensive filtering for high SNR.	Requires calibration to reliably detect damage evolution.	Real-time processing bottlenecks; noise-sensitive DT analysis.

**Table 8:** Key advances of new generation technologies on performance criteria.

Performance	Existing	New Generation	Key Contributions
**Criteria**	**Limitations**	**Technology**	
	**(in Table 7)**		
(C1) SHM Suitability	T2, T3, T5, T7		- Tunnel inspections in GPS-denied environments.
			- Building defects detection after the earthquake.
		(NG1) UAV/Robot	- Real-time, remote, and robust inspection for offshore wind turbine.
		[216,217,218,223,233,238,239,240]	- Automated bridge inspection using UAV-captured images.
			- Robot-based damage assessment for offshore wind turbines.
			- Long-term tunnel inspection using mobile robot.
	T3, T5	(NG2) Self-sensing	- Self-sensing composite wing to achieve structural self-diagnositics.
		[241,242,243]	- Self-sensing UGW to achieve self-health monitoring.
			- Self-sensing CFRP structures by measuring capacitance change.
	T1, T6, T7	(NG3) MSDF [244,245]	- Kalman filtering-based acceleration and displacement fusion.
			- Vibration data from a three-story frame for DL data fusion.
	T1, T2, T7	(NG4) DT 2.0 [246,247,248]	- Customizing monitoring systems for specific conditions.
			- Predictive maintenance through digital modeling of SHM system
			- Decision-making by visualizing structural and operational impacts.
	T2, T5	(NG5) IoT [249,250]	- Continuous monitoring with IoT wireless sensors.
			- Real-time data collection for quick issue response.
			- Enhanced SHM data management
(C2) Hardware	T3, T6	(NG1) UAV/Robot	- Design software development kit for UAV.
Requirements		[216,218,238,239,240]	- Control algorithm designed for UAV-stabilized operation.
			- Agisoft PhotoScan software for the photogrammetric process.
			- A wireless data transmission for quasi-real-time image display.
			- Requirement for 3D LiDAR and an inertial measurement unit.
	T2, T7	(NG2) Self-sensing	- Integrating PZT, strain, temperature, and pressure sensors into the tested wing.
		[241,242,251]	- Waveform generator and signal amplifier used for data collection.
			- Data acquisition circuit and communication module need to be designed.
	T3, T4, T6	(NG3) MSDF	- Consumer-grade camera replacing high-speed camera for displacement measurement.
		[244,245]	- Commercial accelerometers for measurement.
	T3, T4	(NG4) DT 2.0	- High-performance computing for simulating complex scenarios.
		[248,252,253]	- Assist SHM for robust and scalable sensing procedures.
	T2, T6	(NG5) IoT	- IoT streamlines data collection, reducing complexity and cost.
			- Event-triggered sensing extends battery-operated devices’ lifespan.
		[249,254]	- Hardware durable enough to withstand harsh conditions.
(C3) Signal	T1, T4, T6	(NG1) UAV/Robot	- Photogrammetric for high-accuracy point cloud generation.
Characteristics		[238,239,240]	- Stereo camera alignment to decrease reconstruction error.
			- Effective health indicators extracted from 3D point cloud data.
	T3	(NG2) Self-sensing	- Low-pass filtered PZT signals for identifying model parameters.
		[241,242]	- Wave mode identification for captured signals.
	T5, T6	(NG3) MSDF	- Fusion of multi-type sensor data sampled at different rates.
		[244,245]	- Data preprocessing applied before fusion step.
	T3, T4, T5	(NG4) DT 2.0	- Integrating sensor data with historical metrics to enhance accuracy.
		[255,256,257]	- Dynamically adapting models with incoming data to boost accuracy.
			- LiDAR and virtual reality enhance inspection accuracy.
	T1, T4, T7	(NG5) IoT	- Advanced signal processing for managing large data volumes.
		[250,258,259]	- Optimizing data transmission for timeliness and bandwidth balance.
			- Edge computing reduces latency and server dependence.
(C4) Features	T1, T3, T7	(NG1) UAV/Robot	- Less than 2% vertical error for the UAV flying route.
Sensitivity		[216,217,239]	- The synthetic dataset quality impacts the detection accuracy.
			- 5.10% of the mean error for crack size inspection.
	T2	(NG2) Self-sensing	- Dynamic parameter identification using the autoregressive model.
		[241,242,243]	- Linear time-of-flight feature extraction based on collected signals.
			- The titania filler within the epoxy matrix enhances sensor sensitivity.
	T1	(NG3) MSDF [244]	- Measurement noise was estimated to be around 10% of the measured responses.
	T3, T7	(NG4) DT 2.0	- Precise and targeted scenario analyses.
			- Detecting subtle changes through high-fidelity models.
		[260,261,262]	- DL adapts to new degradation and failure patterns.
			- Analyzing the impact of environmental changes.
	T5, T6	(NG5) IoT	- Advanced sensors designed to detect a wide range of parameters.
		[263,264,265]	- Enhancing detection sensitivity with ML algorithms.
			- Remote calibration of actuators and sensors.
(C5) Digital Twin	T2, T4, T5	(NG1) UAV/Robot	- Can be explored by establishing a 3D point cloud model.
Simulation		[216,218,238,239,240]	- Wind turbine models can be setup in an FE environment.
			- The 3D model of dam structure can be constructed.
			- The damage projected in 3D space for damage localization.
			- Reconstruct tunnel model in a virtual environment.
	T3	(NG2) Self-sensing	- The composite wing can be integrated into a DT environment.
		[241,242]	- Composite and wave propagation can be integrated in FE simulation.
	T4, T5	(NG3) MSDF	- The tested structural and virtual sensing can be built in a DT environment.
		[244,245]	- The structure and acceleration signals can be captured in a DT environment.
	T3, T4, T6, T7	(NG4-5) DT 2.0, IoT	- Integration of real-time environmental data into simulation models.
		[266,267]	- Improving simulation accuracy with real-world data.
